# Disruption of super-enhancer-driven tumor suppressor gene RCAN1.4 expression promotes the malignancy of breast carcinoma

**DOI:** 10.1186/s12943-020-01236-z

**Published:** 2020-08-08

**Authors:** Rong Deng, Jun-Hao Huang, Yan Wang, Li-Huan Zhou, Zi-Feng Wang, Bing-Xin Hu, Yu-Hong Chen, Dong Yang, Jia Mai, Zhi-Ling Li, Hai-Liang Zhang, Yun Huang, Xiao-Dan Peng, Gong-Kan Feng, Xiao-Feng Zhu, Jun Tang

**Affiliations:** 1 State Key Laboratory of Oncology in South China, Collaborative Innovation Center for Cancer Medicine, Guangdong Key Laboratory of Nasopharyngeal Carcinoma Diagnosis and Therapy, Sun Yat-sen University Cancer Center, Guangzhou, China; 2 Department of Breast Oncology, Sun Yat-sen University Cancer Center, Guangzhou, China

**Keywords:** Super-enhancer, RCAN1.4, RUNX3, BRD4, Breast carcinoma, Malignancy, Tumor suppressor

## Abstract

**Background:**

Super-enhancers (SEs) play a crucial role in cancer, which is often associate with activated oncogenes. However, little is known about how SEs facilitate tumour suppression. Individuals with Down syndrome exhibit a remarkably reduced incidence of breast cancer (BC), moving the search for tumor suppressor genes on human chromosome 21 (HSA21). In this study, we aim to identify and explore potential mechanisms by which SEs are established for tumor suppressor RCAN1.4 on HSA21 in BC.

**Methods:**

In silico analysis and immunohistochemical staining were used to assess the expression and clinical relevance of RCAN1.4 and RUNX3 in BC. Function experiments were performed to evaluate the effects of RCAN1.4 on the malignancy of breast carcinoma in vitro and in vivo. ChIP-seq data analysis, ChIP-qPCR, double-CRISPR genome editing, and luciferase reporter assay were utilized to confirm RUNX3 was involved in regulating RCAN1.4-associated SE in BC. The clinical value of co-expression of RCAN1.4 and RUNX3 was evaluated in BC patients.

**Results:**

Here, we characterized RCAN1.4 as a potential tumour suppressor in BC. RCAN1.4 loss promoted tumour metastasis to bone and brain, and its overexpression inhibited tumour growth by blocking the calcineurin-NFATc1 pathway. Unexpectedly, we found RCAN1.4 expression was driven by a ~ 23 kb-long SE. RCAN1.4-SE^distal^ was sensitive to BRD4 inhibition, and its deletion decreased RCAN1.4 expression by over 90% and induced the malignant phenotype of BC cells. We also discovered that the binding sites in the SE region of RCAN1.4 were enriched for consensus sequences of transcription factor RUNX3. Knockdown of RUNX3 repressed the luciferase activity and also decreased H3K27ac enrichment binding at the SE region of RCAN1.4. Furthermore, abnormal SE-driven RCAN1.4 expression mediated by RUNX3 loss could be physiologically significant and clinically relevant in BC patients. Notably, we established a prognostic model based on RCAN1.4 and RUNX3 co-expression that effectively predicted the overall survival in BC patients.

**Conclusions:**

These findings reveal an important role of SEs in facilitating tumour suppression in BC. Considering that the combination of low RCAN1.4 and low RUNX3 expression has worse prognosis, RUNX3-RCAN1.4 axis maybe a novel prognostic biomarker and therapeutic target for BC patients.

## Background

Breast cancer is the most common cancer and the leading cause of cancer death in women. Although the overall survival rate for breast cancer patients has improved obviously, there were still 2,088,849 new cases and 626,679 people who died of breast cancer worldwide in 2018, accounting for 11.6% of new cancer cases and 6.6% of cancer-related deaths in 36 kinds of tumours [1]. Breast cancer consists of a group of biologically and molecularly heterogeneous diseases originated from the breast, with carrying complex genetic, epigenetic, and environmental factors in the individual patient [2, 3]. Therefore, novel causative genes and molecular pathways underlying breast cancer progression and metastasis need to be identified and validated.

Super-enhancers (SEs) are large clusters of transcriptional enhancers, including populations of transcription factors (TFs), cofactors, chromatin regulators, and transcription apparatus occupying super-enhancers, that drive the expression of genes that define cell identity [4]. At super-enhancers, TFs trigger the recruitment of chromatin-modifying enzymes to establish a stereotypical pattern of covalent histone modifications on adjacent nucleosomes, such as histone H3 lysine 27 acetylation (H3K27ac) and histone H3 lysine 4 mono-methylation (H3K4me1) [5–7]. Accumulating evidence shows that breast cancer cells generate super-enhancers at oncogenes during tumour pathogenesis. ChIP-seq data analysis reveals a super-enhancer at the ESR1 gene, which encodes oestrogen receptor alpha, only in ER-positive cell line MCF-7 cells but not in normal breast epithelium cells [8]. Whereas triple-negative breast cancer cells rely on a specific gene cluster of oncogenic TFs driven by SEs to **s**ustain proliferation and survival [9]. In addition, Dominik et al. identify 33 hotspots of large (> 100 kb) tandem duplications, a mutational signature associated with homologous-recombination-repair deficiency, are enriched in breast-specific ‘super-enhancer’ regulatory elements [10]. Paola et al. report TNF-NFKB1 signalling pathway directly regulates CD47 by interacting with a constituent enhancer located within a CD47-associated SE specific to breast cancer, which drive CD47 overexpression to escape immune surveillance [11]. SEs are also involved in the aromatase inhibitor drug resistance of breast cancer cells [12]. However, there is little known about how super-enhancers are established for tumour suppression in breast cancer.

Down syndrome (DS), which has a number of characteristic dysmorphic features and congenital or acquired medical problems, is a genetic disorder caused by full or partial trisomy of HSA21. Numerous epidemiological studies demonstrate the significantly lower incidence of nearly all solid tumours in individuals with DS, which lead to the speculation of possible tumor suppressor genes on HSA21 [13, 14]. In this study, we analyzed DS-related genes located on chr 21q22, which is reported to be associated with the main features of DS [15], to determine the genes differentially expressed between tumor tissues and adjacent noncancerous breast tissues in 112 BC patients using TCGA database. Regulator of calcineurin 1 (RCAN1) was identified as one of the most downregulated HSA21 genes in BC patients. However, the precise regulatory mechanisms and functions of RCAN1 in breast cancer are still unclear. Here, we reveal an unexpected epigenetic antitumor mechanism in which RUNX3-mediated SE-driven the expression of RCAN1.4, one of RCAN1 transcripts, to install BC-suppressive programs.

## Methods

### Analyses of TCGA data

The GDC TCGA Breast Cancer cohort Counts and FPKM format RNA-seq data of available invasive breast carcinoma and normal breast tissues were downloaded from UCSC Xena Browser (https://xenabrowser.net/datapages/) in August 2018. The aligned GDC TCGA Breast Cancer cohort clinical survival information was also downloaded from UCSC Xena Browser (https://xenabrowser.net/datapages/) at the same time for further analysis. To analyze the expression of these 344 DS-related genes located on chr 21q22 in 112 pairs breast cancer and adjacent noncancerous breast tissues, we calculated the fold change and adjusted *P*-value of the Counts matrix via the DESeq2 package, in which fold change > 2.0 or fold change < − 2.0, and adjusted *P* value < 0.05 were considered to denote a differentially expressed gene. Then we replotted the heatmap of 10 downregulated genes (FPKM values) with R package “pheatmap”, with the gene expression values centered and scaled in the row direction. To assess the RCAN1 mRNA expression in different breast cancer molecular subtypes, patients were stratified according PAM50 subtypes as previously reported [16]. To assess the combination effect of RUNX3 and RCAN1.4 mRNA levels on disease prognosis using the TCGA database, survival analysis was conducted by the “survival” package in R. We traversed all possible threshold combinations of RCAN1.4 and RUNX3 mRNA expression values to find the best cutoff which can distinguish survival significantly. Finally, the samples that FPKM values of RCAN1.4 mRNA expression <=3.52 and RUNX3 mRNA expression <=3.56 were assigned as “RCAN1.4 low and RUNX3 low” group, while the remaining were categorized as “Other” group.

### CRISPR-Cas9-mediated gene disruption

For CRISPR-Cas9-mediated RCAN1.4 knockout in breast cancer cells, two specific sgRNA sequences 5′-GTTTGCCACACAGGCAATCA-3′ and 5′-GATATCACTGTTTGCCACAC-3′ targeting human RCAN1.4 gene were cloned to LentiCRISPR (pXPR_001) plasmid separately. The packaging plasmids were co-transfected with two pXPR-RCAN1.4 sgRNAs into HEK293T cells using Lipofectamine 2000 (Invitrogen), and viral particles were harvested at 48 h post-transfection. MDA-MB-231 and BT549 cells were infected with viruses for 24 h in the presence of polybrene (8 μg/ml). The positive cells were selected under puromycin for 3 days, and seeded at subcloning density. Knockout clones were identified by immunoblot and sequencing. LentiCRISPR (pXPR_001) plasmid was used as a negative control.

### Double-CRISPR genome editing

To design CRISPR constructs for super enhancer deletion, the sequence 5′-TGGCTGGGAAACCGGCAATG-3′ targeting left flanking super enhancer of human RCAN1.4, and the sequence 5′-GGGGCTGAGTAGAATGGGCG-3′ targeting right flanking super enhancer of human RCAN1.4 were cloned to LentiCRISPR (pXPR_001) plasmid separately. LentiCRISPR (pXPR_001) plasmid was used as a negative control. To confirm the deletion efficiency, SE-spanning PCR primers were designed which flank the outside of the CRISPR sgRNAs and amplify a ~ 20 kb + region. Given efficient CRISPR cutting and repair of DNA through non-homologous end joining, a ~ 320 bp product was expected. The following were the RCAN1.4^distal^ PCR deletion-spanning primers used: F:5′-AGGGGATCACCTGTCTGTGT-3′; R: 5′-TCCTGACCACAGG TGATCCG-3′.

### Dual-luciferase reporter assay

The luciferase reporter was co-transfected with a pRL-TK plasmid into cells by using Lipofectamine 2000 (Invitrogen) in triplicate as previously described [17]. Luciferase activity was measured using the Dual-Glo Luciferase Assay system (Promega) according to the manufacturer’s guidelines. Firefly luciferase activity was normalized to renilla luciferase to control for cell number and transfection efficiency.

#### Chromatin Immunoprecipitation quantitative real-time PCR(ChIP-qPCR)

ChIP was performed using the ChIP assay kit (Cat #53008, Active Motif, USA) according to the manufacturer’s instruction. Cells were cross-linked with 1% formaldehyde at room temperature for 10 min, and then neutralized with glycine for 5 min. Cells were rinsed with ice-cold PBS twice and scraped into 1 ml of ice-cold PBS. Cells were resuspended in SDS lysis buffer and sonicated. After centrifugation, supernatants were collected and diluted in IP dilution buffer. Anti-BRD4 (Cat#13440S, Cell Signaling Technology), anti-H3K27ac (GTX128944, Genetex), anti-H3K4me1 (Cat #5326, Cell Signaling Technology) or control IgG (Cat #2729S, Cell Signaling Technology) was used for immunoprecipitation. After immunoprecipitation, protein A-Sepharose was added and incubated for another hour. Precipitates were washed, and DNA was purified after de-crosslinking for qRT-PCR. All qRT-PCR reactions were done in triplicates. Primers used are listed below: RCAN1.4-Promoter ChIP P1:5′-TCCTTCTTGAGCTGGTGCTT-3′, RCAN1.4-Promoter ChIP P2: 5′-ACAGGATGCTGTGGAAGCTG-3′; RCAN1.4-SE ChIP P1: 5′-AACATGAGTCAGTCAGCACCA-3′, RCAN1.4-SE ChIP P2: 5′-GAACGGTTGGCAAATCCTGG-3′.

#### Analysis of ChIP-seq data

ChIP-seq data sets for SUM149, SUM159, SUM159R, SUM1315, and HCC1395 cells, including H3K27ac and BRD4, were obtained from GSE63581. ChIP-seq data sets for MDA-MB-231 cells, including H3K27ac and H3K4me1, were obtained from GSE72141. ChIP-seq data sets for GM12878, H1-hESC, K562, HSMM, HUVEC, NHEK, NHLF and HMEC cells, including H3K27ac, H3K4me1, H3K4me3, and transcription factor binding, were obtained from ENCODE Project. BigWig files of ChIP-seq experiments were used for visualization in IGV.

### Immunohistochemical assay

Sections were submerged into EDTA citrate buffer (pH 6.0 or pH 8.0), and microwaved for antigenic retrieval. Then the slides were incubated with the primary antibody at 4 °C overnight. Primary antibodies for anti-RCAN1 (GTX100245, Genetex), and anti-RUNX3 (GTX60639, Genetex) were used. Normal mouse/rabbite IgG as negative controls were used to ensure specificity. Then the slides were treated by HRP polymer conjugated secondary antibody for 30 min and developed with diamino-benzidine solution (ZSGB-Bio). Nuclei were counterstained with hematoxylin. Image acquisition was performed using a Nikon camera and software. A positive reaction was indicated by a reddish-brown precipitate in the nucleus for RUNX3 expression, and in the cytoplasm for RCAN1.4 expression. Specifically, we adopted a staining index by multiplying the score for the percentage of positive cells by the intensity score (values 0–12), which obtained as the intensity of RCAN1-positive or RUNX3-positive staining (0, no staining; 1,weak; 2,moderate; 3, strong) and the percentage of positive cells (0 = 1 ~ 5%, 1 = 6% ~ 25%, 2 = 26% ~ 50%, 3 = 51% ~ 75%, 4 = 76% ~ 100%). If a composite score was less than 6 (the median value), it was considered as low RCAN1/RUNX3 expression. If a composite score was equal to or greater than 6 was considered as high RCAN1/RUNX3 expression. Kaplan-Meier plots of overall patient survival were stratified by the low or high expression of RCAN1/RUNX3. To analyze the prognostic value of combining RCAN1.4 and RUNX3 protein levels in breast cancer samples, the composite score of RCAN1.4 expression < 6 and RUNX3 expression < 6 were assigned as “RCAN1.4^−^RUNX3^−^” group, RCAN1.4 expression > = 6 and RUNX3 expression < 6 were assigned as “RCAN1.4^+^ RUNX3^−^” group, RCAN1.4 expression < 6 and RUNX3 expression > = 6 were assigned as “RCAN1.4^−^RUNX3^+^” group, RCAN1.4 expression > = 6 and RUNX3 expression > = 6 were assigned as “RCAN1.4^+^RUNX3^+^” group.

### Statistical analysis

Statistical analyses were conducted using GraphPad Prism 8.0.1. (GraphPad, La Jolla, CA, USA) and SPSS 20.0. Survival curves were plotted by the Kaplan-Meier method in SPSS and assessed using the log-rank test, and univariate Cox proportional hazards regression was carried out to identify HR (hazard ratios) and 95% CI (Confidence intervals). Multivariate analysis was used to determine independent prognostic factors using a Cox proportional hazards regression model. The relationship between RUNX3 expression and RCAN1.4 was assessed using Spearman correlation analysis. The results are presented as the mean ± S.D. was analyzed by unpaired Student’s t test or one-way ANOVA with Dunnett’s multiple comparisons test or one-way ANOVA with Tukey’s multiple comparisons test using GraphPad Prism. The box and whisker graphs for the IHC data was analyzed by a non-parametric Mann-Whitney U test. All the statistical tests were two-sided, *P* < 0.05 was considered statistically significant.

### Further applied methods

Additional Cell Culture and Compounds, Luciferase reporter construction, Generation of stable cells using lentiviral infection, Topologically associating domains (TAD) visualization, Cell Migration and Invasion Assay, Tumor Xenografts and Bioluminescence Analysis, In Vivo Tumorigenesis Assay, Quantitative Real-Time PCR, Immunofluorescence staining, SiRNA Transfection, Immunoblot, and Human breast tumour tissue samples were further described in the Additional file 1.

## Result

### Identification of RCAN1.4 as a candidate tumour suppressor in breast cancer

**Fig. 1:**
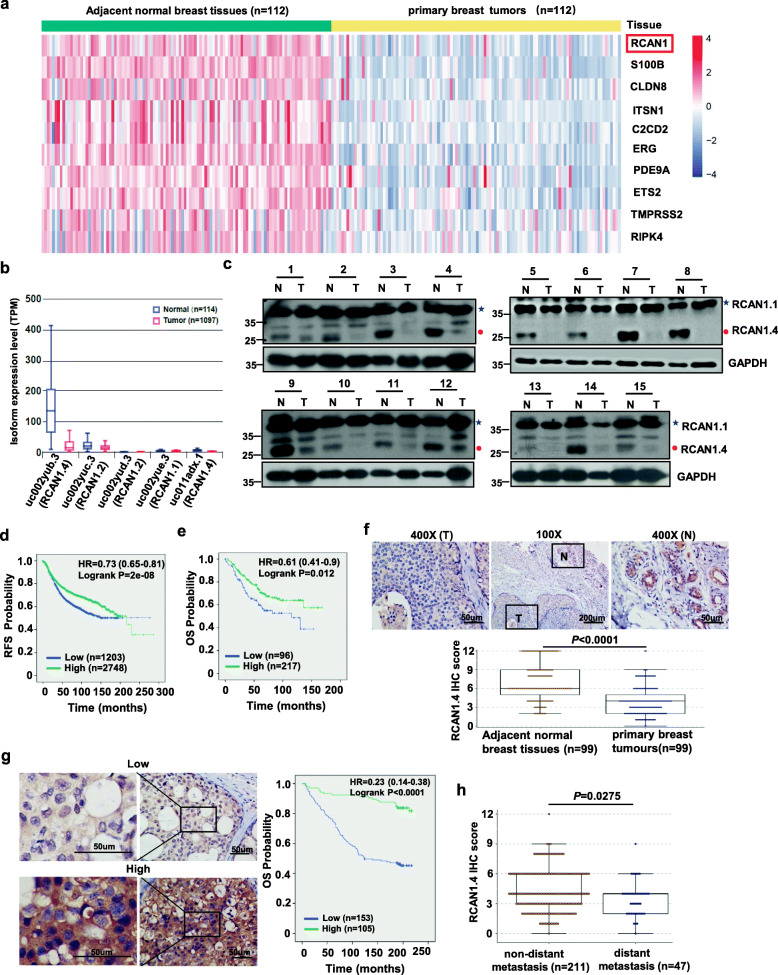
Downregulation of RCAN1.4 and its clinical relevance in breast cancer. **a** The heatmap of 10 downregulated genes located on HSA21 in breast cancer. The FPKM values of 112 pairs of breast cancer invasive samples and adjacent normal samples were retrieved from TCGA database. **b** TCGA analysis showed the expression levels of RCAN1 transcript isoforms in breast cancer tissues and normal tissues using the ISOexpresso website (http://wiki.tgilab.org/ISOexpresso/). **c** Immunoblot results of RCAN1.4 and RCAN1.1 protein expression in 15 pairs of matched breast tissue. Blue star, RCAN1.1; Red closed circle, RCAN1.4. **d-e** Kaplan-Meier analyses of RFS based on RCAN1(215253_at) mRNA levels in all breast cancer patients (d) and Kaplan-Meier analyses of OS based on RCAN1(208370_at) mRNA levels in the breast cancer patients with lymph node positive subgroup (e) were performed by using the KM-plotter breast cancer database (http://kmplot.com/analysis). Auto select best cutoff was chosen to split RCAN1-high and low groups in the analysis. Univariate Cox proportional hazards regression was carried out to identify HR and 95% CI. **f** The representative images of strong RCAN1.4 staining in the matched adjacent normal cells (N) and weak staining in tumor cells (T) (Left). Quantitative IHC analysis of RCAN1.4 staining of primary breast tumours and adjacent normal breast tissues was shown (*n* = 99, right). The *P* value was determined by Wilcoxon matched-pairs signed rank test (two-sided). **g** The representative images for low and high expression of RCAN1.4 staining in 258 primary breast cancer tissues were shown (left). Kaplan-Meier plots of the overall survival of patients, stratified by protein expression of RCAN1.4 (right). **h** The expression of RCAN1.4 in breast cancer tissue without or with distant metastasis. The *P* value was determined by an unpaired non-parametric Mann-Whitney test (two-sided). Error bars in **f**, **h** represented lower hinge - 1.5 * IQR to upper hinge + 1.5 * IQR (where IQR is the inter-quartile range, or distance between the first and third quartiles)

As the risk of breast cancer is decreased among people with DS, we first downloaded chr21q22 gene set from GSEA website, then analyzed the expression of these 344 DS-related genes in 112 pairs of breast cancer and adjacent noncancerous breast tissues from the TCGA database. 21 dysregulated DS-related genes were found (fold change > 2.0 or fold change < − 2.0, adjusted *P* value < 0.05), 10 of which were downregulated (Fig. 1a, Additional file 2: Table S1) and 11 upregulated in BC tissues (Additional file 2: Table S2). RCAN1 was one of the most significantly downregulated HSA21 genes. Further analysis in the TCGA cohort showed that RCAN1 was decreased in different breast cancer molecular subtypes (Additional file 3: Fig. S1a), indicating that downregulation of RCAN1 was a common event in breast cancer patients. As RCAN1 can be expressed as different mRNA isoforms with different functions [18], we then detected the breast cancer-specific RNA expression patterns of isoforms of RCAN1 in the ISOexpresso website. Only three transcripts, including RCAN1.1 (uc002yue.3), RCAN1.2 (uc002yuc.3, uc002yud.3), and RCAN1.4 (uc002yub.3, uc011adx.1), were detected in normal breast tissues and breast cancer tissues. And transcript RCAN1.4 was the major isoform based on the median TPM value and it was significantly decreased up to 85% in breast cancer tissues compared to noncancerous breast tissues (Fig. 1b, Additional file 3: Fig. S1b-S1c). However, the other two transcripts, RCAN1.1 and RCAN1.2*,* were found at very low levels and exhibited no significant difference between the tumour and noncancerous tissues (fold change < 2.0, Fig. 1b, Additional file 3: Fig. S1b-S1c). Downregulation of RCAN1.4 in breast cancer tissues was further confirmed by Western blotting (WB). The results showed that RCAN1.4 expression, but not RCAN1.1 expression, was significantly higher in the matched fresh normal breast tissues than that in tumor tissues (Fig. 1c, Additional file 3: Fig. S1d). These findings suggest that the RCAN1.4 isoform is associated with breast cancer.

Then we further assess the clinical significance of RCAN1 deregulation in breast cancer. The results from Kaplan-Meier meta-analyses using an online database [19] showed that low RCAN1 mRNA expression, especially in the patients with systemically treated, was consistently associated with poor relapse-free survival (RFS) (Fig. 1d, Additional file 3: Fig. S1e-S1f). Also low RCAN1 mRNA expression conferred a shortened overall survival (OS) in the BC patients with lymph node positive (Fig. 1e). Then immunohistochemical (IHC) staining of RCAN1.4 was performed in a cohort of breast primary cancer tissues. The IHC assay showed that the positive staining of RCAN1.4 was mainly observed in the cytoplasm. The expression of RCAN1.4 in the same patient was significantly decreased in tumor tissues compared with that in adjacent noncancerous tissues (Fig. 1f). The Kaplan-Meier survival analysis further revealed that patients with low RCAN1.4 protein expression had a shortened OS compared to those with high expression (Fig. 1g). Statistical analysis also revealed that downregulation of RCAN1.4 correlated with lymph node status, pathological stage and radiotherapy in this cohort (Additional file 2: Table S3). Multivariate Cox regression analyses showed that RCAN1.4 expression status, as well as lymph node status, was an independent prognostic factors of poor OS in BC patients (Additional file 2: Table S4). Moreover, RCAN1.4 expression was lower in BC patients with distant metastasis than those without distant metastasis after treatment (Fig. 1h). Taken together, these findings suggest that low expression of RCAN1.4 is associated with breast cancer progression and is an adverse prognostic marker of survival.

### RCAN1.4 exerts tumour suppressive function in breast cancer by blocking calcineurin-NFATc1 signaling

**Fig. 2:**
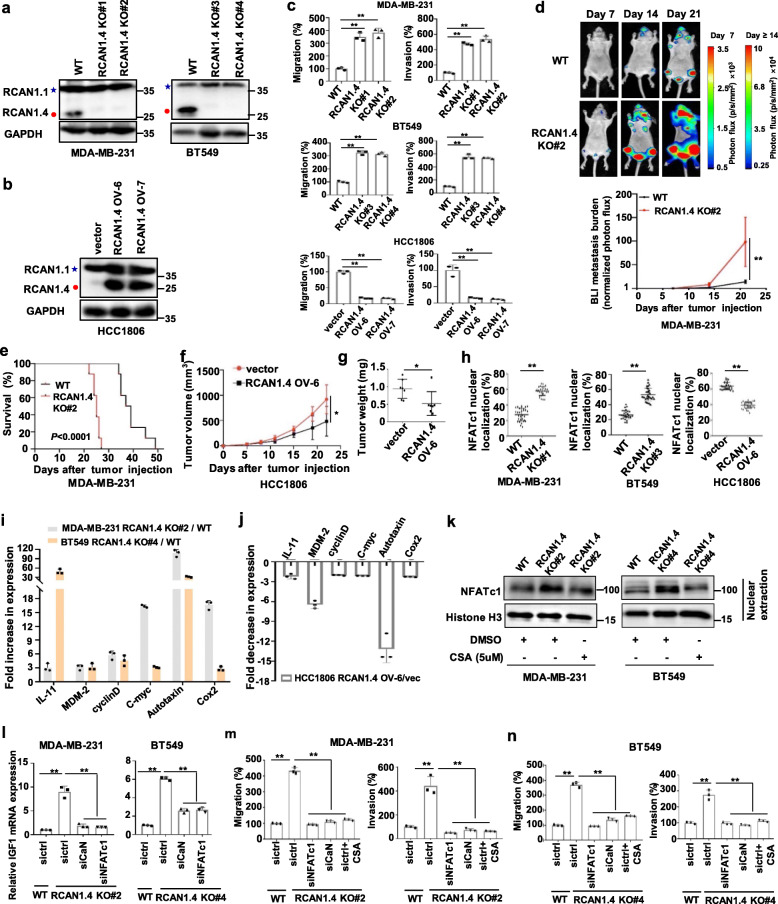
The tumor suppressive effects of RCAN1.4 in breast cancer cells by inhibition of CaN-NFATC1 signaling. **a, b** Immunoblot in MDA-MB-231 and BT549 cells expressing sgRNAs targeting human RCAN1.4 (a), and in HCC1806 cells stably overexpressing RCAN1.4 (b). Blue star, RCAN1.1; Red closed circle, RCAN1.4. **c** The transwell cell migration and invasion assay of the indicated MDA-MB-231, BT549 and HCC1806 cells was shown. The migration or invasion of control cells was set as 100%. **d** 2 × 10^5^ luciferase-tagged WT or RCAN1.4-knockout MDA-MB-231 cells were injected intracardially. BLI images showed representative mice in each group (upper). Normalized metastasis BLI signals from mice (*n* = 8, bottom). **e** Kaplan-Meier survival curve of mice were calculated (*n* = 8). **f-g** Tumour growth of the indicated BT549 cells in BALB/c nude mice (*n* = 7 mice per group). Tumor volumes (f) and tumour weights upon autopsy on day 21(g) were calculated. **h** The indicated MDA-MB-231, BT549 and HCC1806 cells were stained with fluorescent antibodies against NFATc1 (green) or with DAPI (blue). Statistics of the percentage of NFATc1 in the nucleus were determined in the indicated samples (*n* = 30 cells per group). **i-j** qRT-PCR monitoring target gene expression in RCAN1.4-knockout MDA-MB-231 and BT549 cells or RCAN1.4-overexpression HCC1806 cells (*n* = 3 biological independent samples). Expression levels were normalized for GAPDH. Average KO/WT ratios of genes expression in MDA-MB-231and BT549 cells (i), and average OV/vector ratios of genes expression in HCC1806 cells (j) were quantified. **k** The indicated MDA-MB-231 and BT549 cells were treated with 5 μM CsA for 24 h and the nuclear levels of NFATC1 were detected by Immunoblot. **l-n** MDA-MB-231-RCAN1.4 KO cells or BT549-RCAN1.4 KO cells were transfected with CaN siRNAs and NFATC1 siRNAs for 48 h. qRT-PCR was used to monitor IGF1 expression (l, *n* = 3 biological independent samples). Quantification of migratory and invasive cells of images per group was shown (m-n, *n* = 3 biological independent samples). Error bars represent mean ± SD. **P < 0.01. The *P* value in **c** was determined by one-way ANOVA with Dunnett’s multiple comparisons test, the *P* value in **l**, **m**, **n** was determined by one-way ANOVA with Tukey’s multiple comparisons test, no adjustments were made for multiple comparisons. The *P* value in **d**, **f**, **g**, **h** was determined by a two-tailed unpaired Student’s t test. The *P* value in **e** was assessed using the log-rank test. All data are representative of three independent experiments

To investigate the functional role of RCAN1.4 in the progression of breast cancer, we established stable RCAN1.4 knockout models by using specific single-guide RNAs (sgRNAs) in the MDA-MB-231 and BT549 cell lines, as well as a stable RCAN1.4 overexpression model in the HCC1806 cell line, according to the basal RCAN1.4 expression among many breast cancer cell lines (Fig. 2a, b, Additional file 3: Fig. S2a). Also the protein level with RCAN1.4 overexpression in HCC1806 was physiologically relevant (Additional file 3: Fig. S2b). The migration and invasion abilities of RCAN1.4-knockout BC cells were obviously greater than those of wild-type cells (Fig. 2c, Fig.S2c). These abilities decreased when RCAN1.4 was overexpressed (Fig. 2c, Additional file 3: Fig. S2c). Next, we established an experimental animal model of metastasis by intracardiac injection of MDA-MB-231 cells with a stably expressed firefly luciferase reporter. A higher incidence and greater tumour burden were found in mice harbouring RCAN1.4-knockout cells than in mice harbouring wild-type cells, as shown by increased bioluminescence imaging (BLI) signal in the brain and hind limbs (Fig. 2d, Additional file 3: Fig. S2d). More importantly, the survival of mice harbouring RCAN1.4-knockout cells was shortened (Fig. 2e). We also found that overexpression of RCAN1.4 inhibited tumour formation in nude mice, as confirmed by the xenograft tumour volume and tumour weight (Fig. 2f, g, Additional file 3: Fig. S2e). Together, these observations indicate that RCAN1.4 deficiency induces a malignant phenotype in BC cells, promote BC progression and metastasis.

RCAN1.4 has been reported to physically and functionally interact with calcineurin (CaN) and effectively inhibits its phosphatase activity, which could result in specifically blocking calcineurin-mediated NFAT nuclear localization and transcriptional activity [20, 21]. NFAT genes are also involved in the development and metastasis of breast cancer [22]. Therefore, we measured the CaN-NFATc1 signalling in breast cancer cells after stable overexpression or knockout of RCAN1.4. The results showed that knockout of RCAN1.4 promoted nuclear accumulation of NFATc1, whereas overexpression of RCAN1.4 blocked nuclear accumulation of NFATc1 (Fig. 2h, Additional file 3: Fig. S3a). The negative regulation of NFATc1 nuclear translocation by RCAN1.4 was further confirmed by analysis of nuclear extracts (Additional file 3: Fig. S3b). In addition, we measured the expression of known NFATc1 target genes in BC cells [21, 23]. The results showed that knockout of RCAN1.4 significantly increased the mRNA levels of IL-11, MDM-2, cyclin D, c-myc, autotaxin, and Cox2 (Fig. 2i), while overexpression of RCAN1.4 significantly decreased the mRNA levels of these genes (Fig. 2j). We further investigated the possible role of CaN-NFATc1 signalling in RCAN1.4-mediated BC progression. Cyclosporin A (CsA) treatment, a calcineurin inhibitor, reversed the nuclear accumulation of NFATc1 in BC cells, which was upregulated by RCAN1.4 knockout (Fig. 2k), indicating that RCAN1.4 regulated the nuclear localization of NFATc1 by inhibiting calcineurin activity. Also, the mRNA levels of IGF-1, one of the known NFATc1 target genes [21], were significantly increased after RCAN1.4 knockout but rescued by further silencing of NFATc1 and CaN expression (Fig. 2l, Additional file 3: Fig. S3c). Functional assays showed that silencing of calcineurin or NFATc1, as well as treatment with the compound CSA, specifically reduced the migration and invasive activities of BC cells, which were enhanced by RCAN1.4 knockout (Fig. 2m, n, Additional file 3: Fig. S3d, S3e). Taken together, these results suggest that RCAN1.4 can regulate BC progression by acting as an endogenous inhibitor of CaN-NFATc1 signalling.

### RCAN1.4 is a super-enhancer-driven gene

**Fig. 3:**
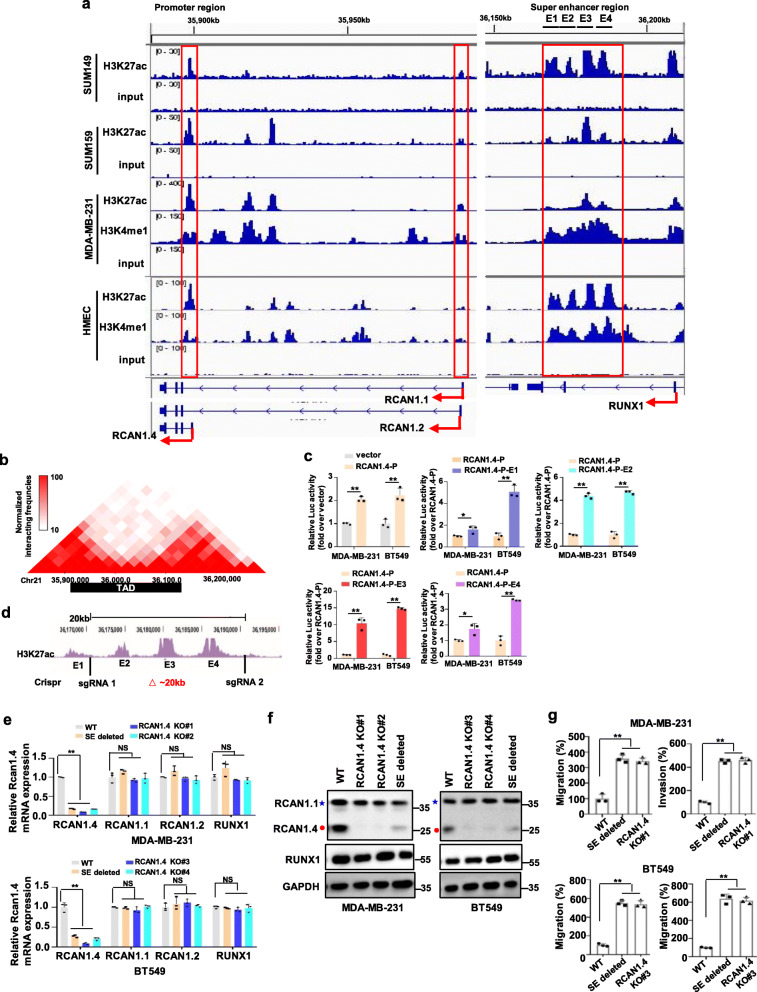
RCNA1.4-SE ^distal^ is responsible for the expression and function of RCNA1.4. **a** Gene tracks depicting the promoter/super-enhancer region of RCAN1.4 and the promoter region of RCAN1.1/RCAN1.2 in mammary epithelial cell HMEC and multiple breast cancer cells with measured H3K27ac, or H3K4me1 marks. The data were retrieved from Encode project (HMEC), GSE63581(SUM149 and SUM159) and GSE72141(MDA-MB-231). **b** RCAN1.4 in HMEC cell line topologically associated domain (TAD) region was predicted on the basis of the Hi-C data (http://promoter.bx. psu. Edu/ hi-c/view.php). **c** The luciferase activities of four enhancer elements were measured through Dual-Luciferase Reporter Assay in MDA-MB-231 and BT549 cells. **d** Schematic representation of deleting distal super-enhancer region of RCAN1.4 strategy. The location of two small guide RNA flanking the 20 kb RCAN1.4-SE^distal^ was shown. **e-f** Deletion of the human composite RCAN1.4 super-enhancer region in MDA-MB-231 and BT549 cells regulated the expression of the indicated genes. The mRNA levels were quantified using qRT–PCR(e). The cell lysates were prepared for immunoblots (f). Blue star, RCAN1.1; Red closed circle, RCAN1.4. **g** Deletion of the human composite RCAN1.4 super-enhancer region affected the migration and invasion ability of MDA-MB-231 and BT549 cells. Quantification of migratory and invasive cells of images per group was shown. Error bars represent mean ± SD, *n* = 3 biological independent samples. NS, not significance, * *P* < 0.05, **P < 0.01. The *P* value in **e**, **g** was determined by one-way analysis ANOVA with Dunnett’s multiple comparisons test, no adjustments were made for multiple comparisons. The *P* value in **c** was determined by a two-tailed unpaired Student’s t test. Data are representative of three independent experiments

To characterize the transcriptional regulation of RCAN1, we examined publicly available ChIP-seq profiles of H3K4me1, H3K27ac and H3K4me3, as well as the corresponding mRNA-seq profiles from the ENCODE (Encyclopedia of DNA Elements) project by using the UCSC Genome Browser. Interestingly, by rank-ordering of enhancer regions within gene-desert regions based on H3K27ac enrichment, we found an approximately 23 kb super-enhancer region of RCAN1.4 (RCAN1.4-SE^distal^, approximately 266 kb downstream of RCAN1.4) in HSMM (Human Skeletal Muscle Myoblasts), HUVEC (Human umbilical vein endothelial cell), NHEK (Normal Human Epidermal Keratinocytes), and NHLF (Normal human lung fibroblasts) cell lines. The active nature of the super-enhancer region in these four cell lines was corroborated by the co-occupancy of both broad H3K4me1 and the supersensitive site of DNA enzyme I (DNase Cluster), which was consistent with the enrichment patterns in the promoter region marked by high H3K4me3, H3K27ac and mRNA peaks (Additional file 3: Fig. S4). While in the GM12878 (human lymphoblastoid), H1-hESC (human Embryonic Stem Cell) and human leukemia K562 cell lines, no peaks appeared in this super-enhancer region, just like the patterns in the promoter region marked by low H3K4me3, H3K27ac and mRNA peaks (Additional file 3: Fig. S4). We also observed that genome-wide landscapes of RCAN1.1 and RCAN1.2 transcripts showed different epigenomic marks from RCAN1.4. The RCAN1.1 transcript and RCAN1.2 transcript had no H3K27ac enrichment in the promoter region and super-enhancer region of these 7 cell lines (Additional file 3: Fig. S4). More important, we observed similar ChIP-seq profiles of H3K27ac and H3K4me1 in mammary epithelial cells (HMECs) and multiple breast cancer cells. The RCAN1.4 transcript, not the RCAN1.1 or RCAN1.2 transcript, was marked by the epigenetic signature of active enhancers H3K27ac and H3K4me1 peaks in the super-enhancer region (Fig. 3a). Furthermore, Hi-C data from HMECs [24] highlighted that the super-enhancer region had direct interactions with the promoter region of RCAN1.4 (Fig. 3b). Next, we further divided the super-enhancer region of RCAN1.4 into four constituents (E1-E4) (Fig. 3a, right), and constructed the RCAN1.4-P (containing the DNA sequences of the RCAN1.4 promoter) to generate the luciferase reporters RCAN1.4-P-E1, RCAN1.4-P-E2, RCAN1.4-P-E3 and RCAN1.4-P-E4 for the luciferase reporter assay. Strong transcription-enhancing activity was observed in MDA-MB-231 and BT549 cells transfected with RCAN1.4-P-E plasmids compared to RCAN1.4-P plasmid, especially those transfected with the RCAN1.4-P-E3 plasmid (Fig. 3c). To test the in vivo function of RCAN1.4-SE^distal^, we sought to delete the super-enhancer sequences (including E2, E3 and E4) from the endogenous locus. We used the CRISPR-Cas9 nuclease system to generate RCAN1.4-SE^distal^ knockout breast cancer cells with deletion of the ~ 20-kb RCAN1.4 composite enhancer by introduction of a pair of sgRNAs (Fig. 3d). Enhancer deletion resulted in near-complete loss of RCAN1.4 mRNA and protein expression to levels similar to those in cells with RCAN1.4 knockout (Fig. 3e, f). Importantly, some other genes on the same chromosomes, such as RCAN1.1, RCAN1.2 and RUNX1, were not directly affected by RCAN1.4-SE^distal^ deletion (Fig. 3e, f), suggesting that RCAN1.4 was indeed the target gene of RCAN1.4-SE^distal^. More important, RCAN1.4-SE^distal^ deletion promoted the migration and invasion abilities in MDA-MB-231 and BT549 cells, as well as the cells with RCAN1.4 knockout (Fig. 3g, Additional file 3: Fig. S5a, S5b). Collectively, these findings indicate that aberrant SE-driven regulation of RCAN1.4 occurs in breast cancer to affect tumour progression.

### RCAN1.4-SE^distal^ is sensitive to BRD4 inhibition

**Fig. 4:**
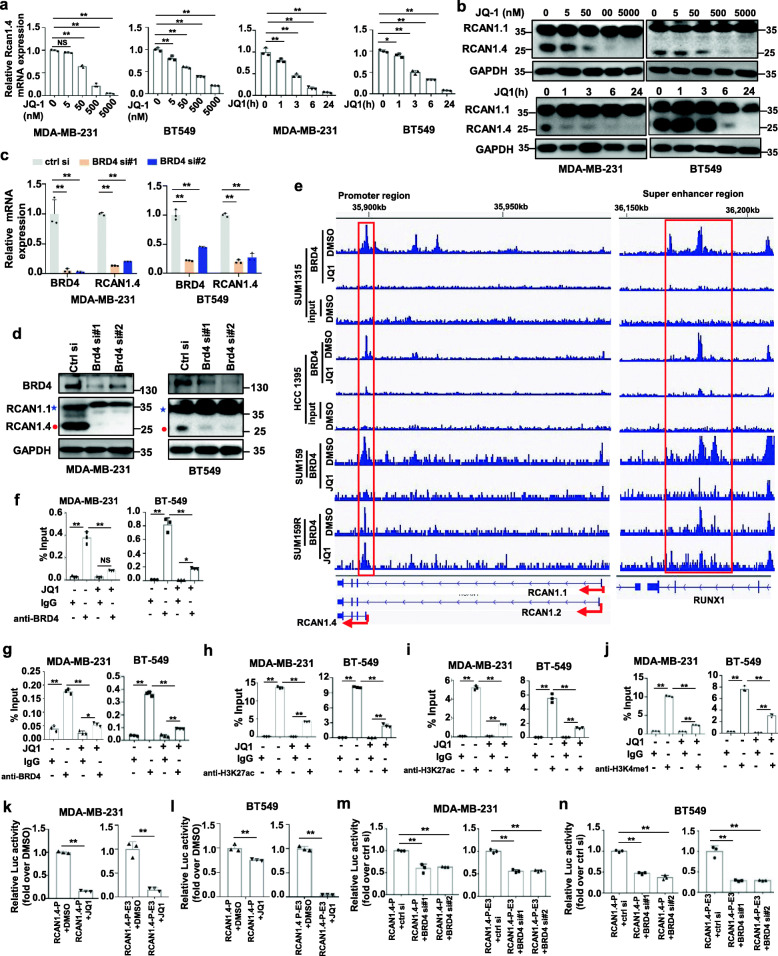
BET inhibition and depletion repress the expression of RCAN1.4. **a**-**b** MDA-MB-231 cells and BT549 cells were treated with various concentrations of BRD4 inhibitor JQ1 for 24 h or 500 nM JQ1 for the indicated times. The RCAN1.4 mRNA levels were quantified using qRT–PCR(a). The cell lysates were prepared for immunoblots (b). **c-d** MDA-MB-231 and BT549 cells were transiently transfected with BRD4 siRNA for 48 h. The RCAN1.4 and BRD4 mRNA levels were quantified using qRT–PCR(c). The cell lysates were prepared for immunoblots (**d)**. Blue star, RCAN1.1; Red closed circle, RCAN1.4. **e** BRD4 binding pattern in the promoter regions and in the SE regions of RCAN1.4. The cells were treated with DMSO or JQ1. The data were retrieved from Gene Expression Omnibus (GSE63581). **f-g** MDA-MB-231 and BT549 cells were treated with or without 200 nM JQ1 for 24 h. The cells were subjected to ChIP analysis using antibodies against BRD4. The association with the SE region (f) and promoter region (g) of RCAN1.4 was quantified by qPCR. An isotype-matched IgG was used as a negative control. **h-i** MDA-MB-231 and BT549 cells were treated with or without 200 nM JQ1 for 24 h. The cells were subjected to ChIP analysis using antibodies against H3K27ac. The association with the SE region (h) and promoter region (i) of RCAN1.4 was quantified by qPCR. **j** MDA-MB-231 and BT549 cells were treated with or without 200 nM JQ1 for 24 h. The cells were subjected to ChIP analysis using antibodies against H3K4me1. The association with the SE region of RCAN1.4 was quantified by qPCR. **k-n** Luciferase reporter assay of RCAN1.4 promote activity and E3 super-enhancer activity in MDA-MB-231 cells and BT549 cells treated with 200 nM JQ1 for 24 h (k, l), or treated with BRD4 siRNA for 48 h (m, n). Error bars represent mean ± SD, n = 3 biological independent samples. * *P* < 0.05, **P < 0.01. The *P* value in **a**, **c**, **m**, **n** was determined by one-way analysis ANOVA with Dunnett’s multiple comparisons test, the *P* value in **f**, **g**, **h**, **i**, **j** was determined by one-way ANOVA with Tukey’s multiple comparisons test, no adjustments were made for multiple comparisons. The *P* value in **k**, **l** was determined by a two-tailed unpaired Student’s t test. Data were representative of three independent experiments

BRD4, one of the bromodomain and extra-terminal domain (BET) protein family members, binds acetylated H3K27 at TFs, TSS, and SEs, brings them together, and mediates transcriptional co-activation and elongation [25]. BET inhibition leads to preferential loss of BRD4 at super-enhancers and specifically diminishes the expression of super-enhancer-driven genes [26, 27]. We further confirmed whether RCAN1.4 expression was driven by BRD4. The result showed that JQ1, a small-molecule inhibitor blocking the binding of BRD4 to H3K27ac, specifically diminished the mRNA and protein levels of RCAN1.4 in a time- and dose-dependent manner (Fig. 4a, b). Also a reduction in RCAN1.4 at the transcriptional and protein levels were observed when BRD4 was knocked down with two independent siRNAs (Fig. 4c, d). However, the mRNA and protein expression of RCAN1.1 and RCAN1.2 did not change after JQ1 treatment or BRD4 knockdown (Fig. 4d, Additional file 3: Fig. S6a-S6c). The ChIP-seq data analysis showed that BRD4 immunoprecipitate shared overlapping enrichment for the promoter and super-enhancer regions of RCAN1.4 in BC cell lines, but this overlap was absent in RCAN1.1 and RCAN1.2 transcripts (Fig. 4e). More importantly, JQ1 treatment reduced the recruitment of BRD4 to the promoter and super-enhancer regions of RCAN1.4 in JQ1-sensitive BC cell lines, but not in JQ1-resistant SUM159R cell line (Fig. 4e). This observation was also validated by our Chip-qPCR analysis with antibodies against BRD4, H3K27ac and H3K4me1. The results showed a significant association of BRD4 with the promoter and super-enhancer region of RCAN1.4, which was decreased with JQ1 treatment (Fig. 4f, g). Additionally, the significant association of H3K27ac with the promoter and super-enhancer region of RCAN1.4 was blocked with JQ1 treatment (Fig. 4h, i), and H3K4me1 levels at super-enhancers in RCAN1.4 were also decreased by JQ1 treatment (Fig. 4j). Next, we detected the effect of JQ1 on the enhancing activity using a luciferase reporter assay. Consistent with ChIP experiments, the luciferase activity of RCAN1.4 promoter and super-enhancer was repressed by JQ1 treatment (Fig. 4k, l). Furthermore, knocking down BRD4 with siRNAs genetically mimicked the effect of JQ1 in repressing the luciferase activity of the RCAN1.4 promoter and super-enhancer (Fig. 4m, n). Collectively, these results confirm that super-enhancer-driven RCAN1.4 expression is susceptible to BRD4 inhibition.

### Super-enhancer-associated RCAN1.4 is transcriptionally activated by RUNX3

**Fig. 5:**
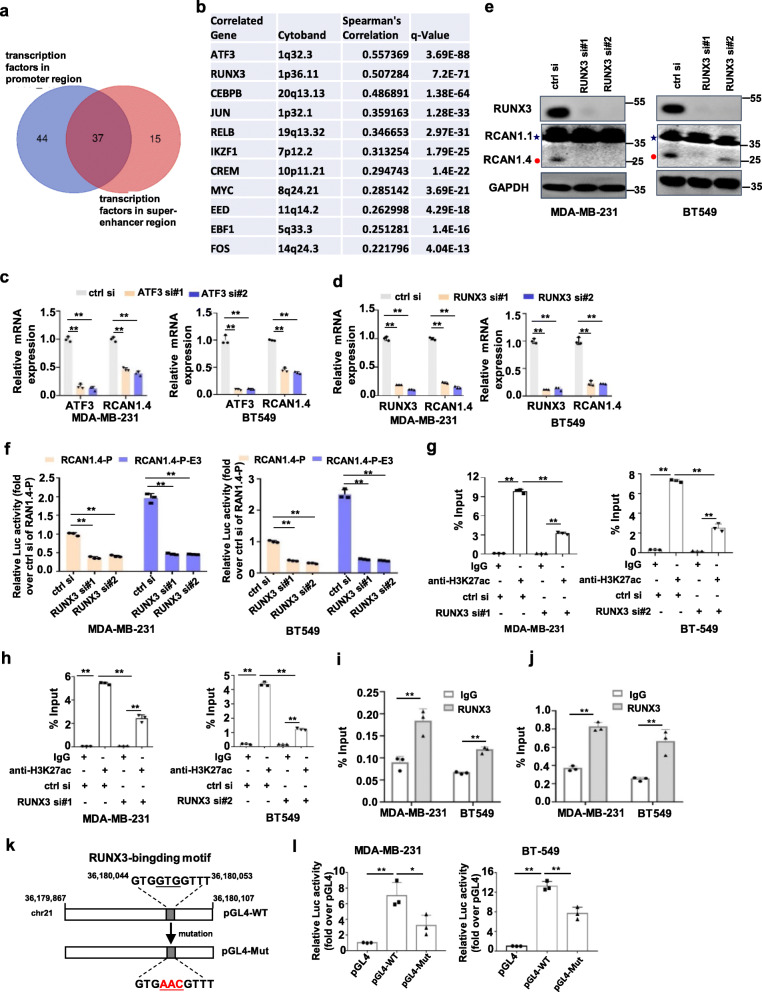
RUNX3 interacts with both the super-enhancer and promoter regions of RCAN1.4 and promotes its transcription. **a** Venn diagrams showing commonly binding transcription factors on the promote and super-enhancer regions of RCAN1.4. **b** The correlation of RCAN1.4 with 11 commonly binding transcription factors on the promoter and super-enhancer regions of RCAN1.4. The data were retrieved from breast invasive carcinoma (TCGA, Firehose Legacy) database using the Cbioportal website (https://www.cbioportal.org/). **c-d** MDA-MB-231 cells and BT549 cells were transfected with siRNAs targeting ATF3 (c) or RUNX3 (d). The mRNA levels were quantified using qRT–PCR. **e** MDA-MB-231 cells and BT549 cells were transfected with siRNAs targeting RUNX3. The cell lysates were prepared for immunoblots. Blue star, RCAN1.1; Red closed circle, RCAN1.4. **f** Luciferase reporter assay of RCAN1.4 promote activity and E3 super-enhancer activity in MDA-MB-231 and BT549 cells with RUNX3 knockdown. **g-h** The MDA-MB-231 and BT549 cells with RUNX3 knockdown were subjected to ChIP analysis using antibodies against H3K27ac. The association with the SE region (g) and promoter region (h) of RCAN1.4 was quantified by qPCR. **i-j** MDA-MB-231 cells and BT549 were subjected to ChIP analysis using antibodies against RUNX3. The association with the promoter region region (i) and SE (j) of RCAN1.4 was quantified by qPCR. **k** Schematic representation of a 241 bp region of the RCAN1.4 enhancer (from chr21:36179867–36,180,107) containing the wild-type RUNX3 motif binding sequence (from chr21:36180044–36,180,053) or the mutant alleles. **l** The MDA-MB-231 and BT549 cells were transfected with the indicated plasmids for 48 h. The levels of luciferase activity were normalized to pRL-TK luciferase activity. Error bars represent mean ± SD, *n* = 3 biological independent samples. * *P* < 0.05, **P < 0.01. The *P* value in **c**, **d**, **f** was determined by one-way analysis ANOVA with Dunnett’s multiple comparisons test, the *P* value in **g**, **h**, **l** was determined by one-way ANOVA with Tukey’s multiple comparisons test, no adjustments were made for multiple comparisons. Data were representative of three independent experiments

We further explored the molecular mechanism by how the super-enhancer regulates the tumour suppressor RCAN1.4 in breast cancer. As enhancers are a class of regulatory DNA elements composed of clusters of transcription factor (TF) binding sites that are uniquely capable of stimulating transcription over large genomic distances [28], we identified candidate transcription factors that could activate RCAN1.4 enhancers. To this end, we analysed the core promoter region and E3 super-enhancer region of RCAN1.4 corresponding to transcription factor binding and H3K27ac H3K4me3 enrichment from previously generated global ChIP-sequencing data of the ENCODE project. Among the 338 transcription factors from the ChIP-seq data, we observed that the core promoter region of the RCAN1.4 locus contained binding sites for 81 transcription factors, overlapping with a region characterized by an enrichment of H3K27ac and H3K4me3(Additional file 3: Fig. S7a). Additionally, we observed that the E3 super-enhancer region of the RCAN1.4 locus contains binding sites for 52 transcription factors, overlapping with a region characterized by an enrichment of H3K27ac and H3K4me1 marks (Additional file 3: Fig. S7b). There were 37 transcription factors binding to both the promoter region and the E3 super-enhancer region of RCAN1.4 (Fig. 5a). Subsequently, we analysed the correlation between RCAN 1.4 and these 37 transcription factors at the transcriptional level in breast cancer patients using the TCGA database, and found ATF3 (Activating Transcription Factor 3) and RUNX3 (RUNX Family Transcription Factor 3) were the top two genes that were most closely and positively correlated with RCAN1.4 in breast cancer tissues (Fig. 5b). Then, we silenced ATF3 and RUNX3 using RNAi. The results showed that RCAN1.4 expression was reduced more potently when RUNX3 was knocked down than that when ATF3 was knocked down (Fig. 5c, d). As RUNX3 is located in 1p36, a region often deleted in breast cancer [29, 30], we focus this study on the regulatory role of this transcription factor. Additionally, the transcriptional levels of RCAN1.1 and RCAN1.2 were not changed when RUNX3 was knocked down (Additional file 3: Fig. S7c). These results were further confirmed at the protein level in MDA-MB-231 and BT549 cells (Fig. 5e).

To clarify the direct regulation of RCAN1.4 by RUNX3 at the transcriptional level through the super-enhancer, we detected whether RUNX3 could regulate the luciferase activity of the RCAN1.4 promoter and super-enhancers. As expected, the luciferase activity of the RCAN1.4 promoter and E3 super-enhancer was repressed when RUNX3 was knocked down (Fig. 5f). We then performed ChIP with an antibody against H3K27ac followed by qPCR. The results showed that RUNX3 loss decreased H3K27ac enrichment at the promoter and super-enhancer regions of RCAN1.4 (Fig. 5g, h). In another direct ChIP-qPCR study, the result showed a significant association of RUNX3 with the promoter and super-enhancer regions of RCAN1.4 (Fig. 5i, j). Analysis of the RCAN1.4-E3 super-enhancer sequences revealed one potential RUNX3 binding site (GTGGTGGTTT) using the JASPAR database (Additional file 3: Fig. S7d). Based on the wild type sequence of the conserved RUNX3-binding sequence (pGL4-WT), we generated a mutant luciferase reporter with the mutant sequence of the conserved RUNX3-binding sequence (pGL4-Mut) (Fig. 5k). The results showed that the luciferase activity of pGL4-WT increased obviously in MDA-MB-231 and BT549 cells, but pGL4-Mut clearly decreased the induction of luciferase activity (Fig. 5l). Taken together, these data indicate that the key transcription factor RUNX3 is required to maintain the super-enhancer activity of RCAN1.4.

### Abnormal SE-driven RCAN1.4 mediated by RUNX3 loss correlates with poor prognosis in breast cancer patients

**Fig. 6:**
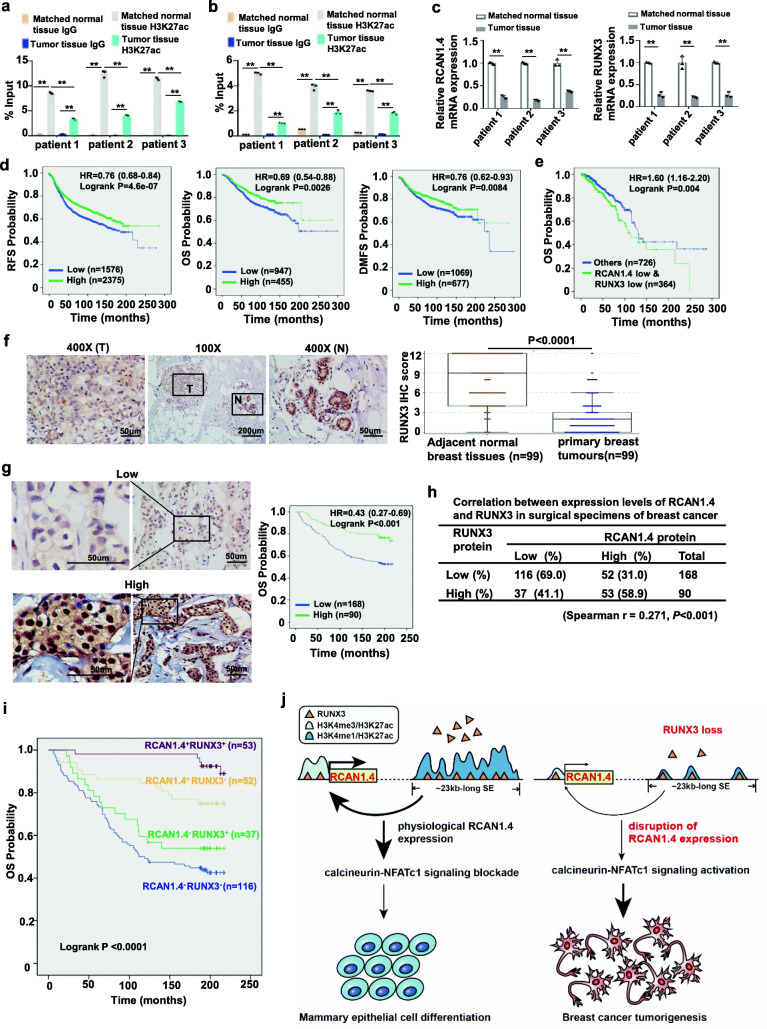
Correlations among RCAN1.4 and RUNX3 expression in breast cancer tissues. **a,b** Three pairs of breast cancer tissues and matched normal breast tissues were subjected to ChIP analysis using antibody against H3K27ac. The association with the supper-enhancer of RCAN1.4 (a) and promoter region of RCAN1.4 (b) were quantified by qPCR. **c** The mRNA expression of RCAN1.4 (left) and RUNX3 (right) in three pairs of breast cancer tissues and matched normal tissues. **d **Kaplan-Meier analyses of OS, RFS and DMFS based on RUNX3 (204197_at) mRNA levels using the KM-plotter breast cancer database (http://kmplot.com/analysis). Auto select best cutoff was chosen in the analysis. **e** Kaplan-Meier plots of the overall survival of patients, stratified by both mRNA expression of RUNX3 and RCAN1.4. The data were retrieved from breast cancer patients of TCGA database**. f** The representative images of strong RUNX3 staining in the matched adjacent normal cells (N) and weak staining in tumor cells (T) (left). Quantitative IHC analysis of RUNX3 staining in primary breast tumors and adjacent normal breast tissues was shown (*n* = 99, right). **g** The representative images for low and high expression of RUNX3 staining in 258 primary breast cancer tissues were shown (left). Kaplan-Meier plots of the overall survival of patients, stratified by protein expression of RUNX3(right). **h** The Spearman correlation of RUNX3 with RCAN1.4 expression in breast cancer patient tumors. **i** Kaplan-Meier plots of the overall survival of patients, stratified by protein expression of RUNX3 and RCAN1.4. **j** Proposed model of RUNX3 deficiency-mediated disruption of SE-driven RCAN1.4 expression to promote the malignancy of breast carcinoma. During the mammary epithelial cell differentiation, the tumor suppressor gene RCAN1.4 expression is driven by a ~ 23 kb-long super-enhancer region, which is located ~ 266 kb away with the epigenetic signature of active enhancers H3K4me1 and H3K27ac. This SE is occupied by the key transcription factor RUNX3 and physically interacts with the RCAN1.4 locus via DNA looping. In breast cancer cells, the loss of RUNX3 results in disruption of super-enhancer-driven RCAN1.4 expression, which promotes the malignancy of breast carcinoma. The error bars represent mean ± SD, n = 3 biological independent samples. **P < 0.01. The *P* value in a, b was determined by one-way ANOVA with Tukey’s multiple comparisons test, no adjustments were made for multiple comparisons. The *P* value in **c** was determined by a two-tailed unpaired Student’s t test. The *P* value in **f** was determined by Wilcoxon matched-pairs signed rank test (two-sided), and the error bars represented lower hinge - 1.5 * IQR to upper hinge + 1.5 * IQR (where IQR is the inter-quartile range, or distance between the first and third quartiles). Data in **a**, **b**, **c** were representative of three independent experiments

Next, we checked H3K27ac levels at the super-enhancer and promoter regions of RCAN1.4 in breast cancer tissues. As expected, ChIP analysis showed a significant association of H3K27ac with the promoter and super-enhancer regions of RCAN1.4 in matched normal breast tissues, which was significantly decreased in the breast cancer tissues (Fig. 6a, b). Accordingly, higher mRNA levels of RCAN1.4 and RUNX3 were observed in normal tissues than in tumour breast tissues (Fig. 6c). To assess the clinical significance of disruption of super-enhancer-driven RCAN1.4 expression mediated by RUNX3 deregulation, we performed Kaplan-Meier meta-analyses using an online database [19]. The results showed that low mRNA expression of RUNX3 was associated with poor OS, RFS, and early distant metastasis-free survival (DMFS) in breast cancer patients (Fig. 6d, Additional file 3: Fig. S8a). Especially in the patients with systemically treated, low RUNX3 mRNA expression was consistently associated with poor OS (Additional file 3: Fig. S8b). We also assessed the combination effect of RUNX3 and RCAN1.4 mRNA levels on disease prognosis using the TCGA database. The breast cancer patients with tumours that had low mRNA expression of both RUNX3 and RCAN1.4 had significantly shorter overall survival than those with tumours that were categorized as “others” (Fig. 6e). We then performed immunohistochemical analysis to evaluate the potential association between RUNX3 and RCAN1.4 protein levels in breast cancer samples. RUNX3 expression in breast carcinoma tissues was significantly lower than that in matched normal breast tissues (Fig. 6f). The Kaplan-Meier survival analysis revealed that the breast cancer patients with low expression of RUNX3 had a shortened OS compared to those with high expression (Fig. 6g). We also observed a significant positive correlation between the expression levels of RUNX3 and RCAN1.4 (Fig. 6h, Additional file 3: Fig. S8c). In addition, we also analysed the prognostic value of combining RCAN1.4 and RUNX3 protein levels in breast cancer samples. The patients with tumours that had both low RUNX3 and RCAN1.4 protein expression had significantly shorter overall survival than those with tumours that had either high expression of RUNX3 or high expression of RCAN1.4 (Fig. 6i). Fisher’s exact test showed that the patients with tumours that had both low RUNX3 and RCAN1.4 protein expression had the higher death rate than those with tumours that had either high expression of RUNX3 or high expression of RCAN1.4 (Additional file 3: Fig. S8d). Altogether, these results suggest that the SE-driven abnormal expression of RCAN1.4 mediated by RUNX3 loss could be physiologically significant and clinically relevant in breast cancer patients.

## Discussion

Numerous epidemiological studies have shown that individuals with Down syndrome exhibit a remarkably reduced incidence of breast cancer. Therefore, determining the specific role that RCAN1 plays in breast cancer is important for tumour prevention and therapy. Our results showed that RCAN1.4 may be an endogenous tumour suppressor of breast cancer and that it played a critical role in the progression and metastasis of breast cancer by blocking CaN/NFATc1 signalling. We unexpectedly discovered that RCAN1.4 was driven by a super-enhancer in breast cancer. We identified a ~ 23 kb-long SE located ~ 266 kb downstream of RCAN1.4 was responsible for driving RCAN1.4 expression and its tumour suppressive function. We also demonstrated that the loss of the key transcription factor RUNX3 resulted in abnormal activity of the distal super-enhancer regulating RCAN1.4 expression, which lead to a decreased RCAN 1.4 expression in BC (Fig. 6j).

RCAN1 has a wide range of biological roles. In individuals with Down syndrome and a mouse model of Down syndrome, the modest elevation in expression afforded by a single extra transgenic copy of RCAN1.4 is sufficient to confer significant suppression of tumour growth in mice and that resistance is a consequence of a deficit in tumour angiogenesis arising from suppression of the VEGF- CaN/NFAT pathway [31]. These findings strongly suggest that the RCAN1.4 may be closely related to the development of tumours, which has been further confirmed by further studies. For instance, RCAN1.4 is downregulated in hepatocellular carcinoma and prevents proliferation, migration, and invasion of cancer cells and growth of orthotopic tumours [21]. However, in contrast, the constitutive overexpression of isoform RCAN1.1, regulated by a different promoter than RCAN1.4, activates NFAT and its proangiogenic activity to promote angiogenesis in vitro [32]. These mean different isoforms of RCAN1 have the opposite functions. Here, we identified RCAN1.4, not RCAN1.1 and RCAN1.2, was the major expression isoform of RCAN1 in normal breast tissues and was significantly decreased in breast cancer tissues. Low RCAN1.4 expression was associated with unfavourable survival. Also RCAN1.4 could act as an independent prognostic marker of survival. In vitro functional assays and in vivo mouse models confirmed that RCAN1.4 knockout promoted tumor metastasis, and RCAN1.4 overexpression inhibited tumour growth. The malignancy depended on calcineurin-mediated NFATc1 nuclear localization in breast cancer cells. Therefore, our study suggests that RCAN1.4 may be a potential tumor suppressor in breast cancer, which at least partially explains the potential mechanism by which individuals with Down syndrome exhibit a remarkably reduced incidence of breast cancer.

The mechanism of RCAN1.4 regulation in cancer is very complicated. Our data presented here provide evidence for a novel molecular mechanism for RCAN1.4 regulation. SEs have been identified as a unique type of transcriptional regulation involved in cancer development [33, 34]. Specifically, this epigenomic signature is often established to activate oncogenes through a variety of mechanisms, including DNA insertion, deletion, translocation, focal amplification, overexpression of an oncogenic transcription factor, and so on [26, 35, 36]. However, the role and the mechanisms by which super enhancers regulate the expression of tumour suppressor genes are not well understood. In the current study, our in silico analysis suggested that cell types characterized by the presence of RCAN1.4 SEs have high levels of RCAN1.4 transcript. This SE physically interacted with the RCAN1.4 locus via DNA looping, and was responsible for over 90% of RCAN1.4 expression, which indicated that RCAN1.4 was indeed the target gene. More importantly, our results also supported the functional significance of RCAN1.4-SE^distal^ in maintaining the malignant phenotype of breast cancer cells. In addition, we elucidated that RCAN1.4 was transcriptionally driven by transcription RUNX3 via a super-enhancer. Emerging evidence indicates that RUNX3 is a tumour suppressor in breast cancer [37]. RUNX3 is frequently inactivated in human breast cancer cell lines and cancer samples by homozygous deletion of the RUNX3 gene, hypermethylation of the RUNX3 promoter, or cytoplasmic sequestration of the RUNX3 protein [37, 38]. As RUNX3 was downregulated in breast carcinoma tissues, our findings explored the reason why super-enhancer-driven RCAN1.4 expression was disrupted in breast cancer. Therefore, although the de novo formation of oncogenic super-enhancers during cellular transformation promotes tumorigenesis, it is likely that RUNX3-mediated SE-driven the expression of RCAN1.4 plays an important role in tumor suppression in normal cells. The finding is consistent with the previous studies, which suggest that regions occupied by super-enhancers are related to both oncogenes and tumor suppressor genes [39–41]. Our study also provides a solid foundation to establish a more effective prognostic model that predicts the overall survival in BC patients based on both RCAN1.4 and RUNX3 expression. In addition, our in silico analyses showed that ATF3 also was a potential candidate transcription factor that binds to the core promoter region and E3 super-enhancer region of the RCAN1.4. As ATF3 is an adaptive-response gene and has a dichotomous role in breast cancer cells in a context-dependent manner [42], the detailed mechanism whether ATF3 is involved in super-enhancer-driven RCAN1.4 expression should be clarified in the further study.

In most of the cancer cases, SEs act as oncogenes to promote tumor growth, which indicates that SEs could be one of the promising therapeutic targets for cancer treatment [34]. Indeed, BRD4 inhibitor has been proved to significantly suppress proliferation and promoted apoptosis in many tumours [43–46], including triple-negative breast cancer [47]. However, it is noteworthy that targeting SEs when using BETi for cancer treatment might cause significant side effects because some tumor suppressor genes will also be suppressed when blocking SEs [34]. Just like in our study, we observed that BRD4 inhibitor JQ1 or knockdown of BRD4 specifically diminished the mRNA and protein levels of tumor suppressor RCAN1.4 in a time- and dose-dependent manner, which at least in part explains why JQ1 could not achieve good anti-tumour effects in solid tumours. Therefore, more studies and better understanding of mechanisms that how SEs facilitate tumour suppression in breast cancer are needed before SEs could be utilized as therapeutic targets.

## Conclusion

To summarize, this study elucidates the precise regulatory mechanisms and functions of RCAN1.4 in breast cancer. We demonstrate a RUNX3-dependent, SE-mediated mechanism for the deregulation of RCAN1.4, which identifies a role for super-enhancers in tumour suppression in breast cancer. Considering that the combination of low RCAN1.4 expression and low RUNX3 expression is shown to have prognostic significance in BC patients, RUNX3-RCAN1.4 axis may serve as a potential prognostic biomarker and therapeutic target.

## Supplementary information

**Additional file 1.** Supplementary material and methods.

**Additional file 2 Table S1.** The 10 downregulated HSA21 genes in the 112 pairs breast Cancer cohort from TCGA database. **Table S2.** The 11 upregulated HSA21 genes in the 112 pairs breast cancer cohort from TCGA database. **Table S3**. Correlation between RCAN1.4 expression and the clinicopathologic characteristics in BC patients. **Table S4.** Effect of factors on overall survival in BC patients in the univariate and multivariate cox regression model. **Table S5.** Primers used in RT-qPCR. **Table S6.** Oligonucleotide sequence of siRNAs. **Table S7. A**ntibodies used in immunoblot.

**Additional file 3 Figure S1.** RCAN1.4 is associated with unfavorable prognosis in BC patients. **Figure S2.** The tumor suppressive effects of RCAN1.4 in BC cells. **Figure S3.** The tumor suppressive effects of RCAN1.4 in BC cells via blocking CaN -mediated NFATc1 nuclear localization. **Figure S4.** ChIP-seq and RNA-seq on the promoter/super-enhancer Region of RCAN1.4 RCAN1.1, and RCAN1.2. **Figure S5.** Deletion of the human composite RCAN1.4 super-enhancer region promotes the migration and invasion ability of BC cells. **Figure S6.** The effect of BET inhibition and depletion on the expression of RCAN1.1 and RCAN1.2. **Figure S7.** RUNX3 activates the transcription of RCAN1.4 by binding to its specific SE. **Figure S8.** RUNX3 is associated with unfavorable prognosis in BC patients. **Figure S9.** Full unedited Western blotting gels for all figures.

## Data Availability

All data generated or analyzed during this study are included in this manuscript.

## Abbreviations

HSA21: Human chromosome 21
DS: Down syndrome
RCAN1: Regulator of calcineurin 1
DSCR1: Down syndrome critical region 1
SEs: Super-enhancers
TFs: Transcription factors
BC: Breast cancer
ChIP: Chromatin Immunoprecipitation
ENCODE: Encyclopedia of DNA elements
H3K27ac: Histone H3 lysine 27 acetylation
H3K4me1: Histone H3 lysine 4 mono-methylation
H3K4me3: Histone H3 lysine 4 tri-methylation
IHC: immunohistochemical
qRT-PCR: Quantitative real-time PCR
TAD: Topologically associating domains
CsA: Cyclosporin A
ATF3: Activating transcription factor 3
RUNX3: RUNX family transcription factor 3
