# Supplementary material for: Disruption of super-enhancer-driven tumor suppressor gene RCAN1.4 expression promotes the malignancy of breast carcinoma
Source: Mol Cancer. 2020 Aug 8;19:122. doi: 10.1186/s12943-020-01236-z (PMC7414732; doi:10.1186/s12943-020-01236-z)
Supplement: Supplementary file 1 — Additional file 1. Supplementary material and methods. [file 12943_2020_1236_MOESM1_ESM.docx]

**Supplementary material and methods**

**Cell Culture and Compounds**

Human MDA-MB-231, MDA-MB-361, HS578T, MDA-MB-468, MCF-7, T47D, ZR75-1, SKBR3, BT474, MDA-MB-453, and MDA-MB-435 were maintained in Dulbecco’s modified Eagle’s medium (DMEM) supplemented with 10% fetal bovine serum (FBS, Gibico) and 1% penicillin/streptomycin at 37 °C under 5% CO_2_. AU565, BT549 and HCC1806 cells were maintained in RPMI-1640 medium supplemented with 10% FBS, and 1% penicillin-streptomycin. All the cells were authenticated using short-tandem repeat profiling, and tested negative for mycoplasma contamination. Compounds cyclosporin A (S2286) and JQ1(S7110) were obtained from Selleck Chemicals.

**Luciferase reporter construction**

For the RCAN1.4-P luciferase reporter, a 1851-bp region of RCAN1.4 promoter (from chr21:35898937-35900813) was subcloned into the XhoI and HindIII sites of pGL3-Basica vector (Promega). To generate the RCAN1.4 P-E luciferase reporter, a 1899-bp region of the RCAN1.4-E1 enhancer (from chr21:36167153-36169051) , a 1838-bp region of the RCAN1.4-E2 enhancer (from 36173677-36175514) , a 1533-bp region of the RCAN1.4-E3 enhancer (from chr21:36179691- 36181223) , and a 1999- bp region of the RCAN1.4 E4 enhancer (from chr21:36185229-36187227) were separately subcloned into RCAN1.4-P luciferase reporter via SalI and BamHI restriction sites , which was downstream of luciferase, to generate the luciferase reporters RCAN1.4-P-E1, RCAN1.4-P-E2, RCAN1.4-P-E3 and RCAN1.4-P-E4 plasmids. To confirm the RUNX3 binding site on the RCAN1.4-E3 enhancer, a 241-bp region (from chr21:36179867-36180107) containing the wild type RUNX3 motif binding sequence was inserted into the SalI and BamHI sites of pGL4.23 vector (Promega Co.,) which was downstream of luciferase and a minimal promoter, to generate a RUNX3 luciferase reporter (pGL4-WT). To generate pGL4-Mut plasmid, the mutated RUNX3 binding sites at nucleotides chr21:36,180,044-36,180,053 from GTGGTGGTTT to GTGAACGTT were constructed by PCR-based site directed mutagenesis.

**Generation of stable cells using lentiviral infection**

Human full-length RCAN1.4 cDNA (with fused myc-tag) was subcloned into were subcloned into PCDH-CMV-MCS-EF1 vector (System Biosciences). For RCAN1.4 overexpression, packaging plasmids were co-transfected with PCDH- RACN1.4 into HEK293T cells, and viral particles were harvested at 48 hr post-transfection. RACN1.4- overexpression cells were infected with viruses for 24 hr in the presence of polybrene (8μg/ml), and stable cells were subsequently selected by puromycin for 3 days. RACN1.4-overexpression clones were isolated by single-cell dilution cloning and validated by immunoblot.

**Tumor Xenografts and Bioluminescence Analysis**

Female BALB/c nude mice were obtained from Sun Yat-sen University, Guangzhou and were 6- to 8-wk-old. All procedures involving mice and experimental protocols were approved by Institutional Animal Care and Use Committee (IACUC) of Sun Yat-sen University Cancer Center. Luciferase-tagged tumour cells were injected into the left cardiac ventricle of anesthetized female nude mice. Development of brain and hind limbs metastases was monitored by measuring photon flux of BLI signals in the hindlimbs of mice after intraperitoneal injection of 75 mg/kg D-Luciferin (PerkinElmer). Bioluminescence images were acquired with the IVIS Imaging System (Xenogen) at 2-5 minutes after injection. BLI signal data were acquired after background subtraction. Data were normalized to the signal obtained immediately after xenografting (day 0).

**Cell Migration and Invasion Assay**

Assays were performed in 24-well Boyden chambers (Corning). Transwell inserts (8 μm pore size) coated with growth factor reduced matrigel (BD Biosciences) for invasion assays. Tumor cells were seeded inside transwell inserts containing 200 ul culture media without FBS. As a chemoattractant, 600 ul culture media containing 10% FBS was placed in the lower chamber. After 18-24 h, cells that translocated to the lower surface of filters were fixed in 4% formaldehyde, stained with 0.1% [crystal](javascript:void(0);) [violet](javascript:void(0);) solution, and counted using a light microscope.

**Quantitative Real-Time PCR**

Total RNA was isolated by TRIzol according to the manufacturer’s instructions. And reverse transcription was performed using a PrimeScript™ RT Reagent Kit with gDNA Eraser (RR047D, Takara). Quantitative Real-Time PCR (qRT-PCR) was conducted using ChamQ SYBR qPCR Green Master Mix (Q311-03, Vazyme Biotech Co.), and run with a Light Cycler 480 instrument (Roche Diagnostics). The relative amount of target gene mRNA was normalized to GAPDH. All qRT-PCR reactions were done in triplicates. The primers used were listed in Additional file 2: Table S5.

***In Vivo* Tumorigenesis Assay**

For *in vivo* tumorigenesis assays, tumor xenografts were established by 2 × 10^6^ tumor cells mixed with matrigel (1:1) injected mammary fat pad into nude mice. Tumor volumes and body weight of mice were observed. Tumor volumes were calculated by the formula: 0.5 × a × b^2^ in millimeters, where a is the length and b is the width. After nude mice were killed, the tumor tissues were excised and weighed.

**Topologically associating domains (TAD) visualization**

RCAN1.4 TAD in [HMEC](https://www.encodeproject.org/) cell line was obtained from [HMEC](https://www.encodeproject.org/) Hi-C interaction data and visualized by Interactive Hi-C Data Browser (http://promoter.bx.psu.edu/ hi-c/ view.php).

**Immunofluorescence staining**

For Immunofluorescence staining, the cells seeded on Glass Bottom culture dishes. The samples were stained with primary antibodies against NFATc1(GTX22796, Genetex) diluted in 4% BSA at 4℃ overnight. The cells were washed, and followed by a fluorescently labeled secondary antibody Alexa Fluor 488-conjugated antibodies against mouse or rabbit IgG (Thermo Scientific), and developed with DAPI. Confocal images were examined using a microscope (Olympus). Quantification was performed using ImageJ software (National Institutes of Health).

**SiRNA Transfection**

The cells were seeded into six-well plates the day before transfection. Transfection of siRNA was performed with lipofectamine RNAimax (Invitrogen) according to the manufacturer’s instruction. The sequences of human siRNAs were listed in Additional file 2: Table S6.

**Immunoblot**

For immunoblot，cells were harvested and lysed in 1xSDS sample buffer or 1×cell lysis buffer (Cell Singnaling) adding 1mM phenylmethanesulfonyl fluoride immediately before use. A volume of 25-50 ug of total proteins was separated by SDS-PAGE transferred to PVDF membrane. After incubated with Primary antibodies, membranes were incubated with horseradish peroxidase (HRP)-conjugated secondary antibody for 1 h and chemiluminescence signals were detected by ECL substrate (Cell signaling). Quantification of Western Blots was performed using ImageJ software. Antibodies used in immunoblot were listed in in Additional file 2: Table S7. Full unedited western blotting gels were shown in Additional file 3: Fig. S9.

**Human breast tumour tissue samples**

To elucidate the mechanism of super-enhancer regulating the tumour suppressor RCAN1.4 in breast cancer, 15 fresh pairs of human breast cancer tissues and matched normal tissues were lysed for Western Blot, ChIP-qPCR or RT-PCR analysis. To elucidate the key role of RCAN1 and RUNX3 during breast cancer progression, 258 paraffin-embedded human breast primary cancer samples (as judged by review of the hematoxylin and eosin–stained sections), were selected for this study. These samples were histopathologically and clinically diagnosed at the Sun Yat-sen University Cancer Center from June 1999 to January 2002. These operable cases selected were based on availability of no distant metastases, resection tissue, follow-up data, and had not received neoadjuvant therapy. All patients were treated consistently according to the same treatment guideline of our center. Clinical and clinicopathologic classification and staging were determined according to the 2002 AJCC pTNM staging system for breast cancer. All samples used in this study were approved by the medical ethics committee of Cancer Center of Sun Yat-sen University. Clinical information on the samples is summarized in Table S3.
