# Supplementary material for: Disruption of super-enhancer-driven tumor suppressor gene RCAN1.4 expression promotes the malignancy of breast carcinoma
Source: Mol Cancer. 2020 Aug 8;19:122. doi: 10.1186/s12943-020-01236-z (PMC7414732; doi:10.1186/s12943-020-01236-z)
Supplement: Supplementary file 2 — Additional file 2 Table S1. The 10 downregulated HSA21 genes in the 112 pairs breast Cancer cohort from TCGA database. Table S2. The 11 upregulated HSA21 genes in the 112 pairs breast cancer cohort from TCGA database. Table S3. Correlation between RCAN1.4 expression and the clinicopathologic characteristics in BC patients. Table S4. Effect of factors on overall survival in BC patients in the univariate and multivariate cox regression model. Table S5. Primers used in RT-qPCR. Table S6. Oligonucleotide sequence of siRNAs. Table S7. Antibodies used in immunoblot. [file 12943_2020_1236_MOESM2_ESM.docx]

**Table S1. The 10 downregulated HSA21 genes in the 112 pairs breast Cancer cohort from TCGA database**

| **Gene Symbol** | **Full Name** | **Tumor/ Normal ratio** | **Adjusted P value** |
| --- | --- | --- | --- |
| S100B | S100 calcium binding protein B | 0.11 | 4.66E-40 |
| CLDN8 | Claudin 8 | 0.29 | 2.24E-10 |
| ITSN1 | Intersectin 1 | 0.32 | 5.63E-41 |
| RCAN1 | Regulatorof calcineurin 1 | 0.33 | 8.57E-28 |
| C2CD2 | C2 calcium dependent domain containing 2 | 0.34 | 2.23E-39 |
| ERG | ETS Transcription Factor ERG | 0.36 | 1.54E-47 |
| PDE9A | Phosphodiesterase 9A | 0.43 | 9.73E-21 |
| ETS2 | ETS proto-oncogene 2, transcription factor | 0.44 | 1.30E-39 |
| TMPRSS2 | Transmembrane protease serine 2 | 0.45 | 1.82E-07 |
| RIPK4 | Receptor interacting serine/ threonine kinase 4 | 0.49 | 7.06E-09 |

**Table S2. The 11 upregulated HSA21 genes in the 112 pairs breast cancer cohort**

**from TCGA database**

| **Gene**  **Symbol** | **Full**  **name** | **Tumor/ Normal ratio** | **Adjusted P value** |
| --- | --- | --- | --- |
| TMPRSS3 | Transmembrane Serine Protease 3 | 2.03 | 1.76E-05 |
| MIS18A | MIS18 Kinetochore Protein A | 2.03 | 2.52E-55 |
| SLC37A1 | Solute Carrier Family 37 Member 1 | 2.14 | 1.73E-42 |
| DONSON | Downstream neighbor of SON | 2.41 | 2.43E-75 |
| CHAF1B | chromatin assembly factor 1 subunit B | 2.41 | 1.64E-39 |
| DOPEY2 | DOP1 Leucine Zipper Like Protein B | 2.56 | 7.43E-54 |
| RSPH1 | Radial Spoke Head Component 1 | 2.74 | 7.61E-18 |
| MX1 | MX dynamin like GTPase 1 | 3.00 | 3.96E-22 |
| CLIC6 | Chloride Intracellular Channel 6 | 4.10 | 1.71E-13 |
| TFF3 | trefoil factor 3 | 4.80 | 2.79E-17 |
| TFF1 | trefoil factor 1 | 6.24 | 4.50E-13 |

**Table S3 Correlation between RCAN1.4 expression and the clinicopathologic**

**characteristics of the breast cancer patients**

| **Patients characteristics** | **No. of cases (%)** | **RCAN1 Expression No.** | | ***P* value** |
| --- | --- | --- | --- | --- |
|  |  | **low** | **High** |  |
| Age (years) |  |  |  | 0.582 |
| ≧40 | 202(78.3) | 118 | 84 |  |
| ＜40 | 56 (21.7) | 35 | 21 |  |
| T classification |  |  |  | 0.117 |
| T1 | 62 (24.0) | 30 | 32 |  |
| T2 | 162 (62.8) | 103 | 59 |  |
| T3 | 34 (13.2) | 20 | 14 |  |
| N classification |  |  |  | 0.038 |
| N0 | 112 (43.4) | 56 | 56 |  |
| N1 | 68 (26.4) | 42 | 26 |  |
| N2 | 34 (13.2) | 23 | 11 |  |
| N3 | 44 (17.1) | 32 | 12 |  |
| Stage |  |  |  | 0.033 |
| I | 35 (13.6) | 15 | 20 |  |
| II | 138 (53.5) | 80 | 58 |  |
| III | 85 (32.9) | 58 | 27 |  |
| Estrogen receptor |  |  |  | 0.873 |
| Negative | 125 (50.6) | 74 | 51 |  |
| Positive | 122 (49.4) | 71 | 51 |  |
| PR |  |  |  | 0.690 |
| Negative | 134 (53.4) | 78 | 56 |  |
| Positive | 117 (46.6) | 71 | 46 |  |
| Her-2 |  |  |  | 0.403 |
| Negative | 189 (73.3) | 115 | 74 |  |
| Positive | 69 (26.7) | 38 | 31 |  |
| Grade |  |  |  | 0.843 |
| 1+2 | 195 (75.9) | 116 | 79 |  |
| 3 | 62 (24.1) | 36 | 26 |  |
| Chemotherapy |  |  |  | 0.562 |
| Yes | 206 (79.8) | 124 | 82 |  |
| No | 52 (20.2) | 29 | 23 |  |
| Radiotherapy |  |  |  | 0.016 |
| Yes | 180 (69.8) | 98 | 82 |  |
| No | 78 (30.2) | 55 | 23 |  |

**Table S4.** **Effect of factors on overall survival in BC patients in the univariate and multivariate cox regression model**

| **Factors** | | **Univariate** | | |  | **Multivariate** | | | |
| --- | --- | --- | --- | --- | --- | --- | --- | --- | --- |
|  |  | **HR** | **95%CI** | ***P* value** |  | | **HR** | **95%CI** | ***P* value** |
| Age (> 40 vs. ≤ 40 years) | 0.825 | | 0.521～1.304 | 0.410 |  | | 0.893 | 0.523～1.523 | 0.677 |
| T stage (T3-T4 vs. T1-T2) | 1.618 | | 0.960～2.725 | 0.071 |  | | 1.084 | 0.595～1.974 | 0.792 |
| N stage (N2-N3 vs. N0-N1) | 2.927 | | 1.977～4.332 | <0.001 |  | | 2.308 | 1.446～3.683 | <0.001 |
| ER (Positive vs. Negative) | 0.766 | | 0.513～1.145 | 0.194 |  | | 1.091 | 0.645～1.845 | 0.746 |
| PR (Positive vs. Negative) | 0.733 | | 0.493～1.091 | 0.126 |  | | 0.744 | 0.441～1.254 | 0.267 |
| Her-2 (Positive vs. Negative) | 1.363 | | 0.896～2.073 | 0.148 |  | | 1.393 | 0.867～2.238 | 0.171 |
| Grade (3 vs. 1+2) | 1.098 | | 0.698～1.726 | 0.687 |  | | 0.928 | 0.562～1.533 | 0.770 |
| RCAN1.4 (high vs. low) | 0.229 | | 0.137～0.383 | <0.001 |  | | 0.244 | 0.144～0.414 | <0.001 |
| Chemotherapy (Yes vs. No) | 0.944 | | 0.584～1.527 | 0.815 |  | | 1.016 | 0.586～1.762 | 0.954 |
| Radiotherapy (Yes vs. No) | 2.274 | | 1.533～3.374 | <0.001 |  | | 1.393 | 0.859～2.259 | 0.180 |

**Table S5. Primers used in RT-qPCR**

| **Gene** | **Forward (5’-3’)** | **Reverse (5’-3’)** |
| --- | --- | --- |
| RCAN1.4 | TTTAGCTCCCTGATTGCCTGT | AAAGGTGATGTCCTTGTCATACG |
| BRD4 | AGCAGCAACAGCAATGTGAG | GCTTGCACTTGTCCTCTTCC |
| IL-11 | GGGGACATGAACTAGGGACA | GGTAGGACAGTAGGTCCGCT |
| c-MYC | TACAACACCCGAGCAAGGAC | AGCTAACGTTGAGGGGCATC |
| MDM2 | CTTGGCCTGGGTTACATGGT | CACGGAGCTTGAGAGGAAGT |
| cyclinD1 | TTACAGTAGCGTAGCGTGCC | AGCGTATCGTAGGAGTGGGA |
| Autotaxin | GGCCGGTGTACCCAACTAAA | AAATTTCTCTCGCCCTCGCA |
| COX2 | TGAGCATCTACGGTTTGCTG | TGCTTGTCTGGAACAACTGC |
| GAPDH | CGCTGAGTACGTCGTGGAGTC | GCTGATGATCTTGAGGCTGTTGTC |
| RCAN1.1 | TGGAGCTTCATTGACTGCGA | ACGTCCTAAAGAGGGACTCA |
| RCAN1.2 | GGCGACGTGACTCAGTGTTC | GGTGATGTCCTTGTCATACGTC |
| RUNX1 | CCTCAGGTTTGTCGGTCGAA | CTGCCGATGTCTTCGAGGTT |
| RUNX3 | GGCAATGACGAGAACTACTCCG | GATGGTCAGGGTGAAACTCTTCC |
| ATF3 | GACCAACCATGCCTTGAGGA | GGATGGCAAACCTCAGCTCT |

**Table S6. Oligonucleotide sequence of siRNAs**

| **Gene Name** | **Sequence** |
| --- | --- |
| CaN siRNA(human) | 5'- CACCATGTCTGGGAGATGGAA -3' |
| NFATc1 siRNA(human) | 5'- GAGUCUCUCAGUUCAGUGU -3' |
| RUNX3 si#1(human) | 5’-CCUUCAAGGUGGUGGCAUUTT-3’ |
| RUNX3 si#2(human) | 5’-GUG AUGGCAGGCAAUGACGAGAAC-3’ |
| Brd4 si#1(human) | 5′-TGGCGTTTCCACGGTACCAAA-3′ |
| Brd4 si#2(human) | 5’-AAGACAAAGAAGGGAGUGA-3‘ |
| ATF3 si#1(human) | 5'-UUCUCCGAACGUGUCACGUTT-3' |
| ATF3 si#2(human) | 5'-GCAAAGTGCCGAAACAAGA-3' |
| RCAN1.1 (human) | 5'-GCGAGAUGGAGGAGGUGGAC-3' |
| RCAN1.4 (human) | 5'-CUGUGUGGCAAACAGUGAU-3' |

**Table S7. Antibodies used in immunoblot**

| **Antibody** | **Company** | **Catalog number** |
| --- | --- | --- |
| RCAN1 | Santa Cruz | sc-377507 |
| RUNX3 | ABGENT | AP14667c |
| NFATc1 | Genetex | GTX22796 |
| PPP3CB | proteintech | 66614-1 |
| BRD4 | Santa Cruz | sc-48772 |
| RUNX1 | ABGENT | AP21105a-1 |
| GAPDH | Cell Signaling Technology | 5174 |
| Anti-mouse IgG, HRP-linked | Cell Signaling Technology | 7076 |
| Anti-rabbit IgG, HRP-linked | Cell Signaling Technology | 7074 |
