# Supplementary material for: Disruption of super-enhancer-driven tumor suppressor gene RCAN1.4 expression promotes the malignancy of breast carcinoma
Source: Mol Cancer. 2020 Aug 8;19:122. doi: 10.1186/s12943-020-01236-z (PMC7414732; doi:10.1186/s12943-020-01236-z)
Supplement: Supplementary file 3 — Additional file 3 Figure S1. RCAN1.4 is associated with unfavorable prognosis in BC patients. Figure S2. The tumor suppressive effects of RCAN1.4 in BC cells. Figure S3. The tumor suppressive effects of RCAN1.4 in BC cells via blocking CaN -mediated NFATc1 nuclear localization. Figure S4. ChIP-seq and RNA-seq on the promoter/super-enhancer Region of RCAN1.4 RCAN1.1, and RCAN1.2. Figure S5. Deletion of the human composite RCAN1.4 super-enhancer region promotes the migration and invasion ability of BC cells. Figure S6. The effect of BET inhibition and depletion on the expression of RCAN1.1 and RCAN1.2. Figure S7. RUNX3 activates the transcription of RCAN1.4 by binding to its specific SE. Figure S8. RUNX3 is associated with unfavorable prognosis in BC patients. Figure S9. Full unedited Western blotting gels for all figures. [file 12943_2020_1236_MOESM3_ESM.docx]

**Supplementary figures and legends**


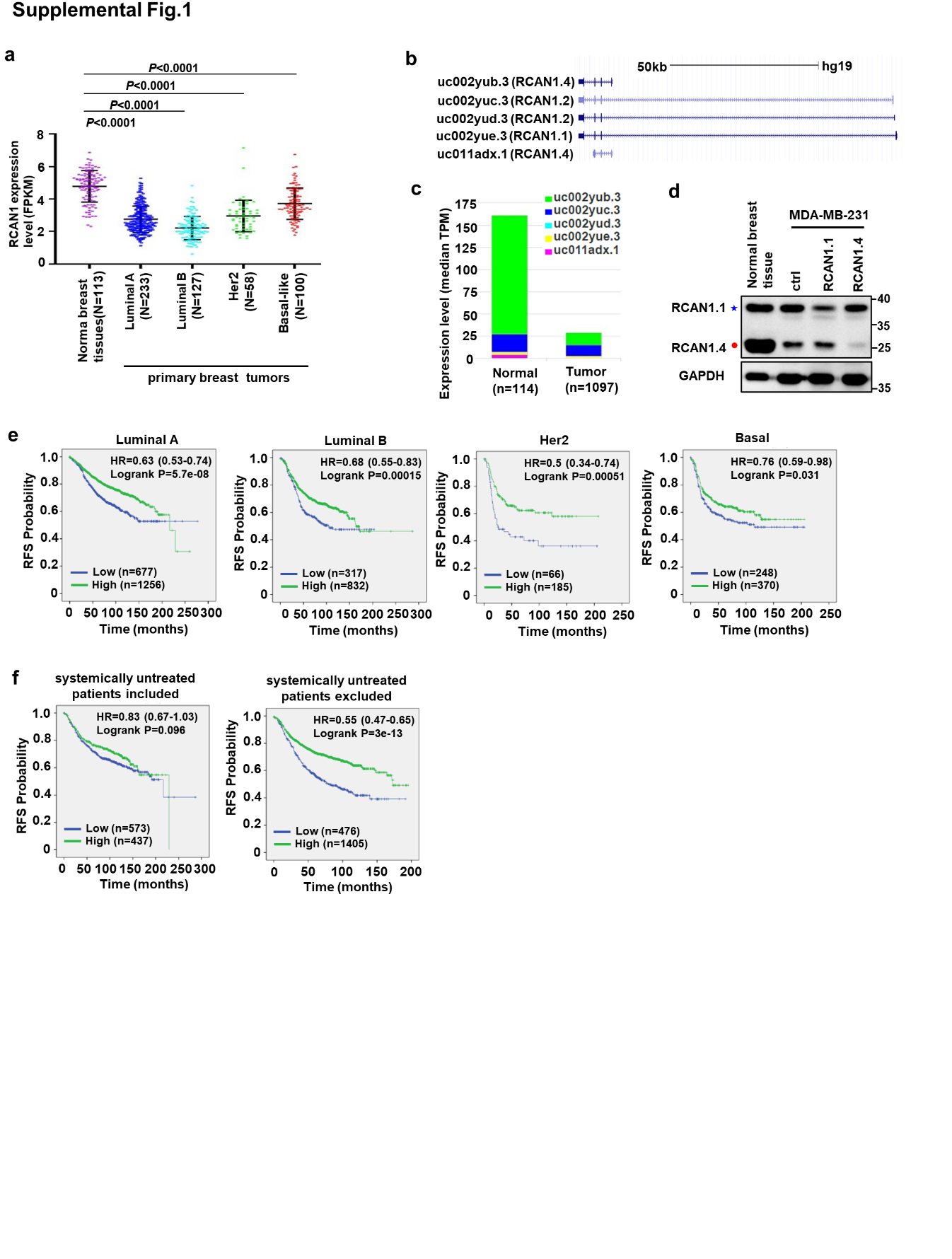


**Figure S1.** **RCAN1.4 is associated with unfavorable prognosis in BC patients. (a)** TCGA analysis showed the expression levels of RCAN1 across different subtypes of breast cancers compared to that in normal breast tissues. Patients were stratified according PAM50 subtypes as indicated. The *P* value was determined by one-way analysis ANOVA with Dunnett’s multiple comparisons test, no adjustments were made for multiple comparisons. **(b)** Schematic representation of transcript isoforms of RCAN1 in the ISOexpresso website (http://wiki.tgilab.org/ISOexpresso/). **(c)** TCGA analysis showed the expression levels of RCAN1 transcript isoforms in breast cancer tissues and normal tissues using the ISOexpresso website (<http://wiki.tgilab.org/> ISOexpresso/). **(d)** MDA-MB-231 cells were transiently transfected with siRNAs specifically targeting RCAN1.1 or RCAN1.4 for 48 h. The expression of RCAN1.1 and RCAN1.4 were detected by WB. Blue star, RCAN1.1; Red closed circle, RCAN1.4. **(e-f)** Kaplan-Meier analyses of RFS based on RCAN1(215253_at) mRNA levels were performed using the KM-plotter breast cancer database (<http://kmplot.com/analysis>). Auto select best cutoff was chosen in the analysis. Univariate Cox proportional hazards regression was carried out to identify HR and 95% CI. The patients were stratified according intrinsic subtype as indicated (e). The patients were stratified according the systemically untreated patients included or excluded (f).


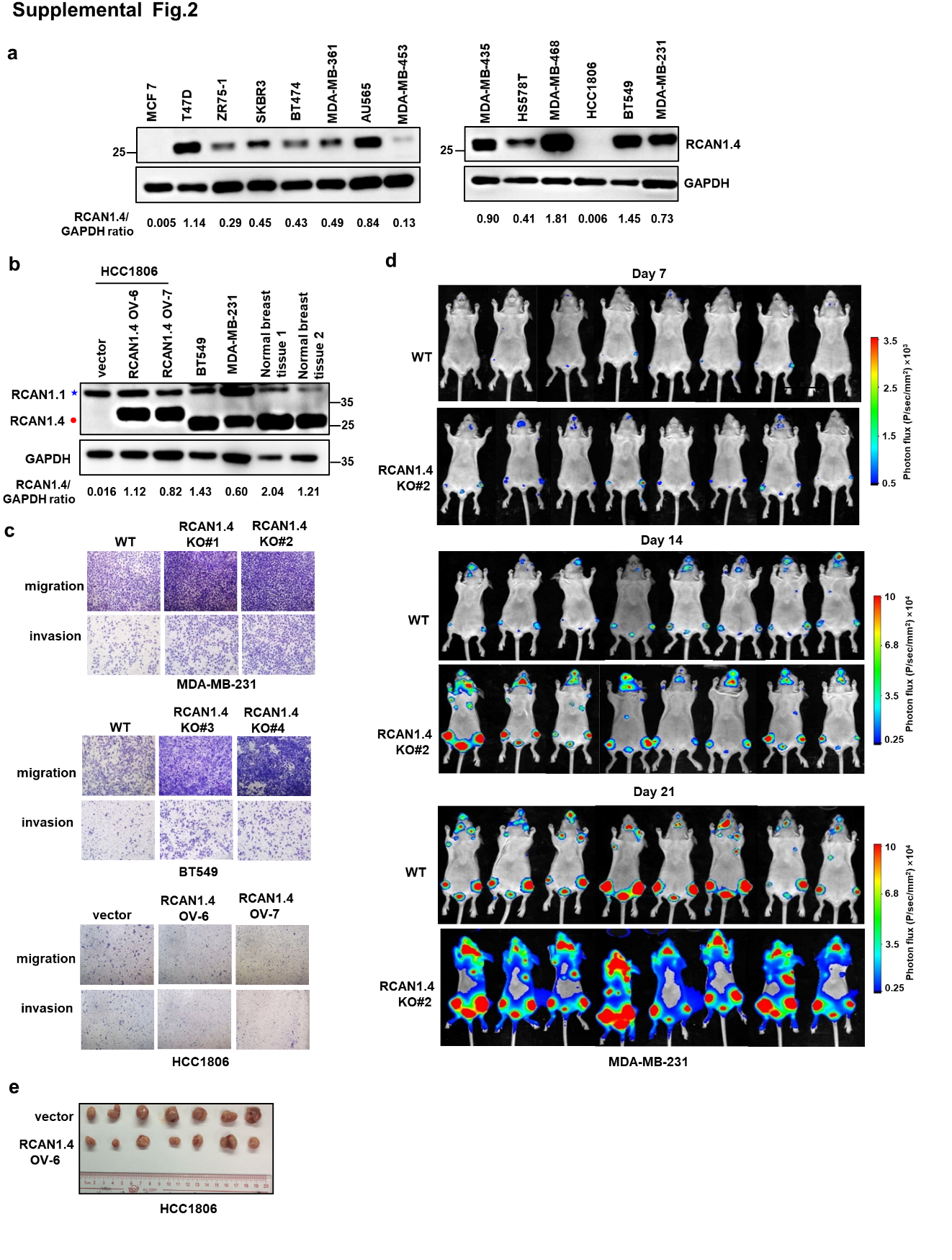


**Figure S2.** **The tumor suppressive effects of RCAN1.4 in BC cells. (a)** The protein expression of RCAN1.4 in 14 breast cancer cell lines including luminal cell lines (MCF-7, T47D, and ZR75-1), HER2-positive cell lines (SKBR3, BT474, MDA-MB-361, AU565 and MDA-MB-453), and TNBC cell lines (MDA-MB-435, HS578T, MDA-MB-468, HCC1806, BT549, and MDA-MB-231). **(b)** The expression of RCAN1.1 and RCAN1.4 in the indicated breast tumor cells and two normal breast tissues were detected by WB. Blue star, RCAN1.1; Red closed circle, RCAN1.4. **(c)** Representative images of the migratory and invasive ability of the indicated MDA-MB-231 cells, BT549 and HCC1806 cells. **(d)** 2×10^5^ the indicated luciferase-tagged MDA-MB-231 cells were injected intracardially. BLI images showed all mice from each experimental group. **(e)** The images showed all excised tumor tissues when mice were killed on day 22. The Data are representative of three independent experiments.


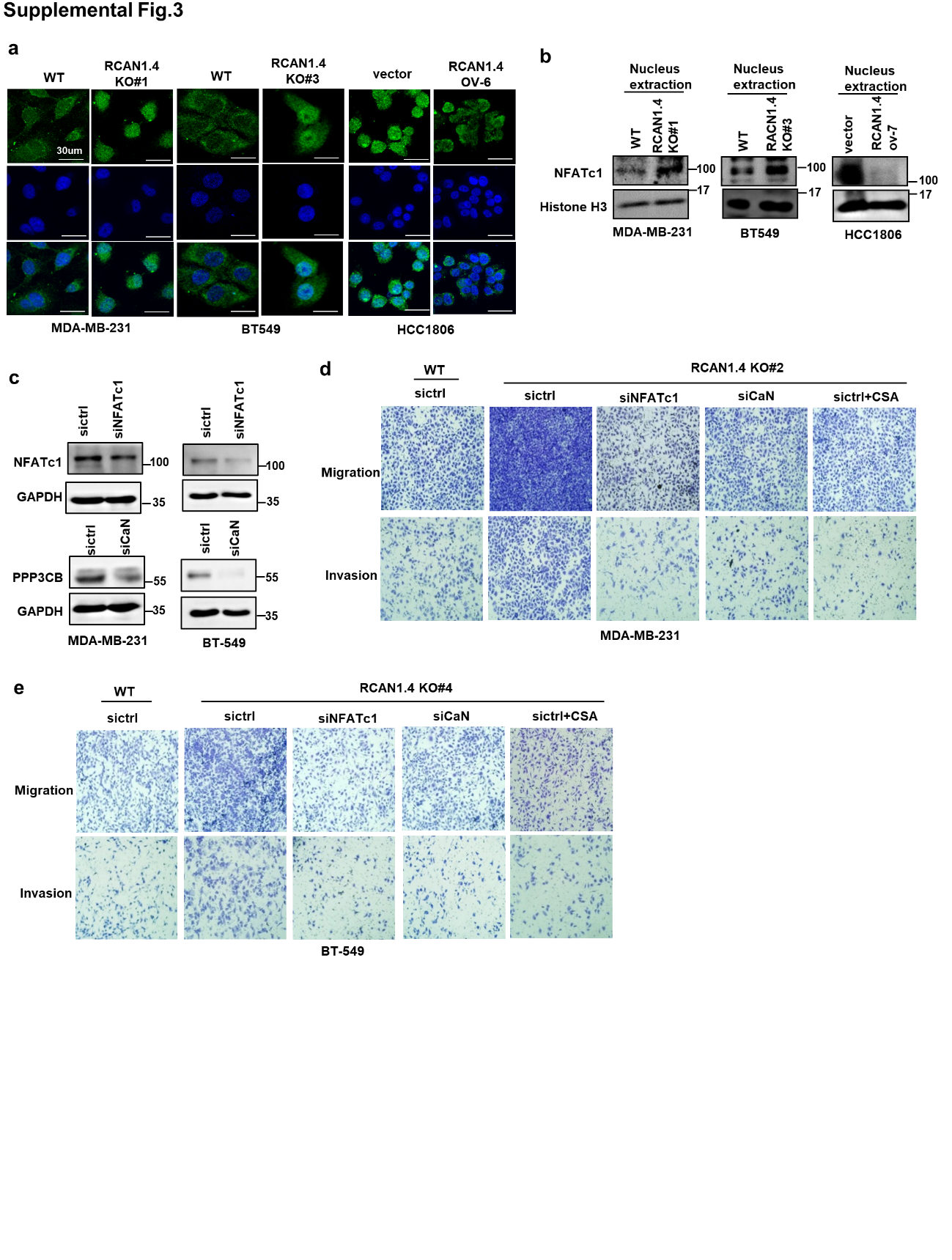


**Figure S3.** **The tumor suppressive effects of RCAN1.4 in BC cells via blocking CaN-mediated NFATc1 nuclear localization.** **(a)** The indicated MDA-MB-231, BT549 and HCC1806 cells were stained with fluorescent antibodies against NFATc1 (green) or with DAPI (blue). Representative confocal immunofluorescence images were shown. **(b)** Nuclear levels of NFATc1 in the indicated BC cells were detected by WB. **(c)** Immunoblot analysis of MDA-MB-231 and BT549 cells transfected with the NFATc1 and CaN siRNA oligonucleotides for 48h. **(d-e)** MDA-MB-231 KO cells or BT549 KO cells were transfected with CaN siRNAs and NFATc1 siRNAs for 48h, or were treated with 5μM CsA for 24h. Representative images of the migratory and invasive ability of the indicated MDA-MB-231(d) and BT549(e) cells. Data are representative of three independent experiments.

**
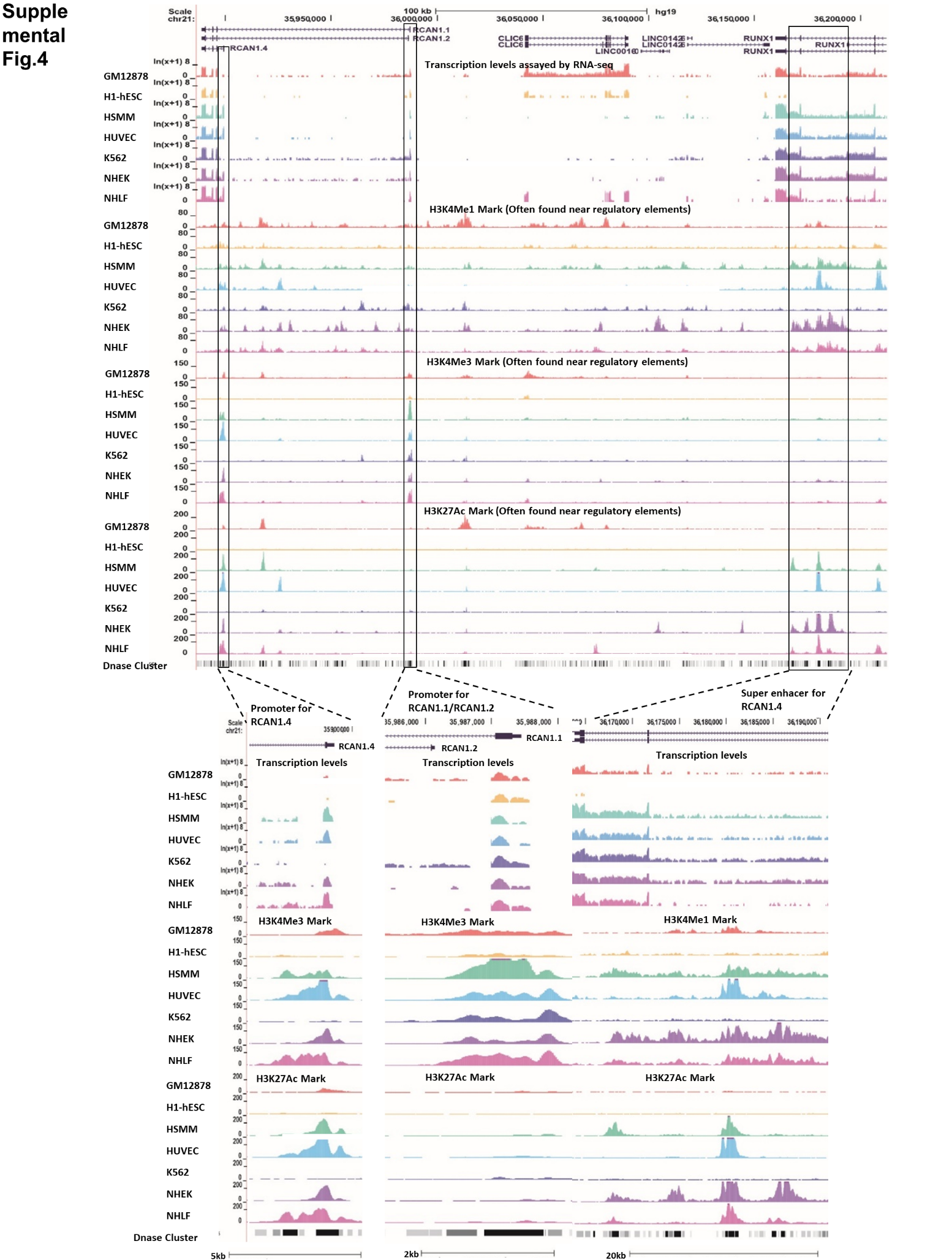
Figure S4.** **ChIP-seq and RNA-seq on the promoter/super-enhancer Region of RCAN1.4, RCAN1.1, and RCAN1.2**. Visualization of the promoter/enhancer region of RCAN1.4 and the promoter region of RCAN1.1/RCAN1.2 in seven cell lines with measured H3K4me1, H3K4me3, and H3K27Ac marks and transcription levels as compiled by the UCSC Genome Browser on Human GRCh37/hg19 Assembly.

**
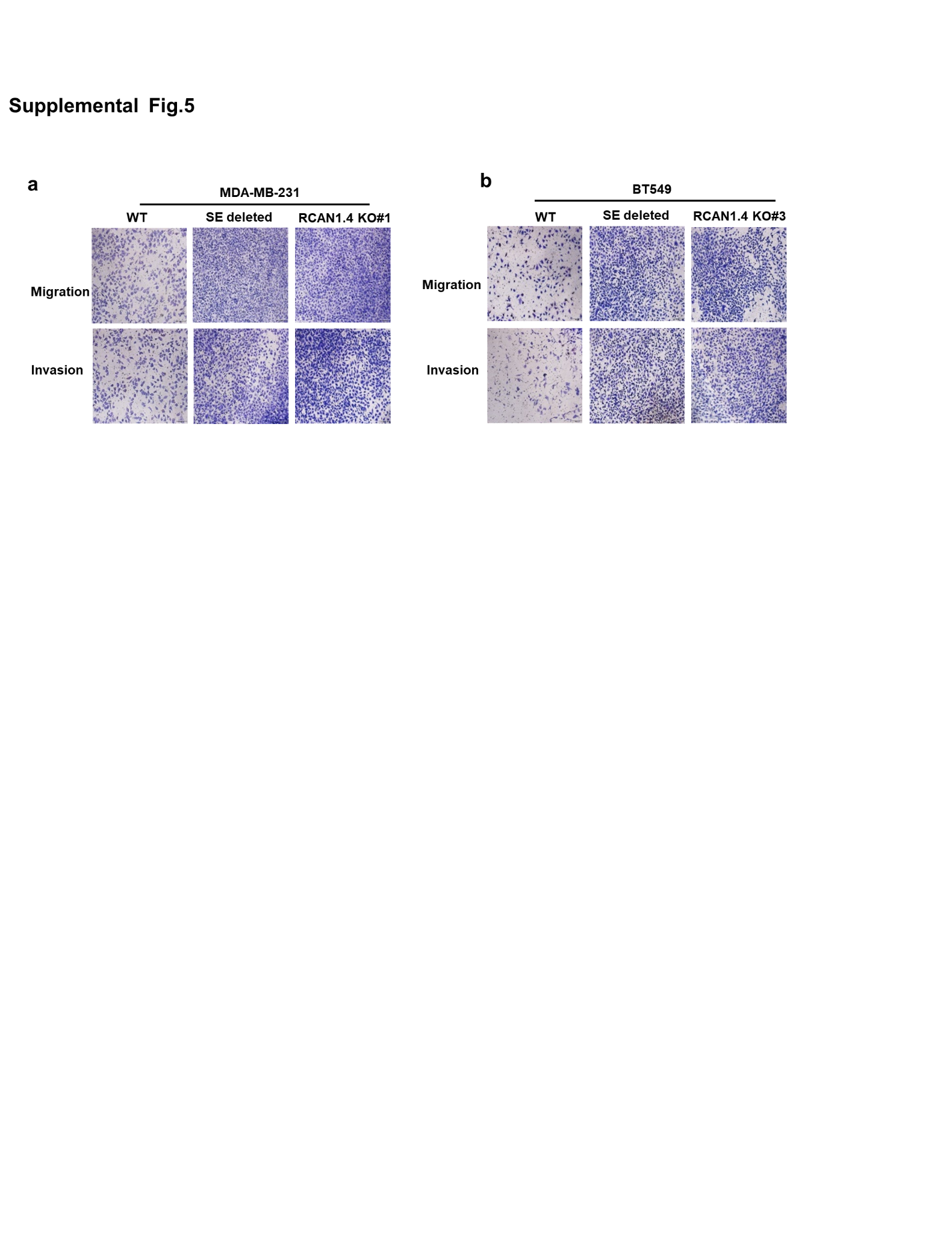
**

**Figure S5.** **Deletion of the human composite RCAN1.4 super-enhancer region promotes the migration and invasion ability of BC cells. (a-b)** Representative images of the migratory and invasive ability of the indicated MDA-MB-231(a) and BT549 (b) cells. Data are representative of three independent experiments.

**
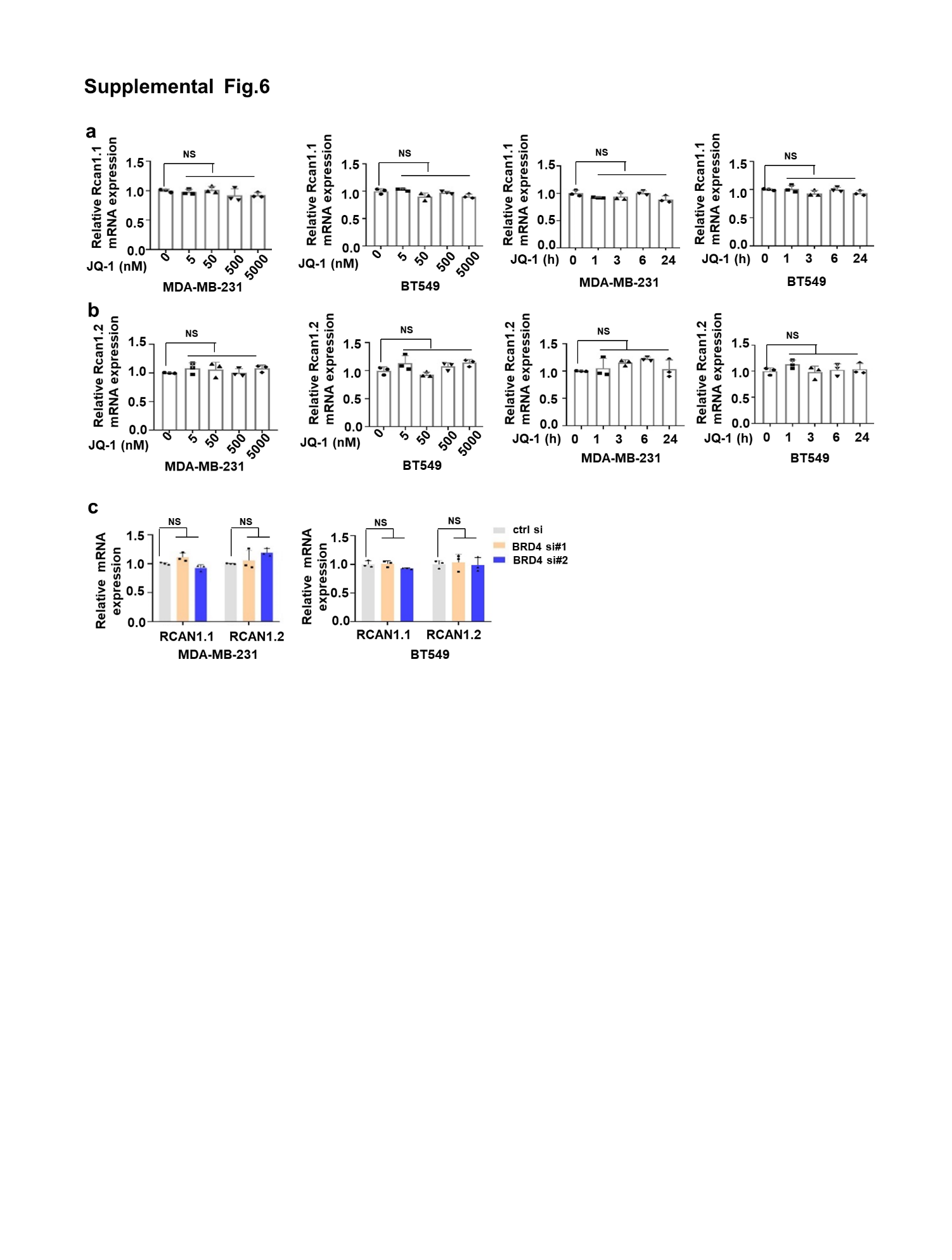
Figure S6.** **The effect of BET inhibition and depletion on the expression of RCAN1.1 and RCAN1.2. (a-b)** MDA-MB-231 and BT549 cells were treated with various concentrations of BRD4 inhibitor JQ1 for 24h or 500nM JQ1 for the indicated times. The RCAN1.1 mRNA levels (a) and RCAN1.2 mRNA levels (b) were quantified using qRT–PCR. **(c)** MDA-MB-231 and BT549 cells were transiently transfected with BRD4 siRNA for 48 h. The RCAN1.1 and RCAN1.2 mRNA levels were quantified using qRT–PCR. Error bars represent mean ± SD, n=3 biological independent samples. NS, not significance. The *P* value was determined by one-way analysis ANOVA with Dunnett’s multiple comparisons test, no adjustments were made for multiple comparisons. Data were representative of three independent experiments.

**
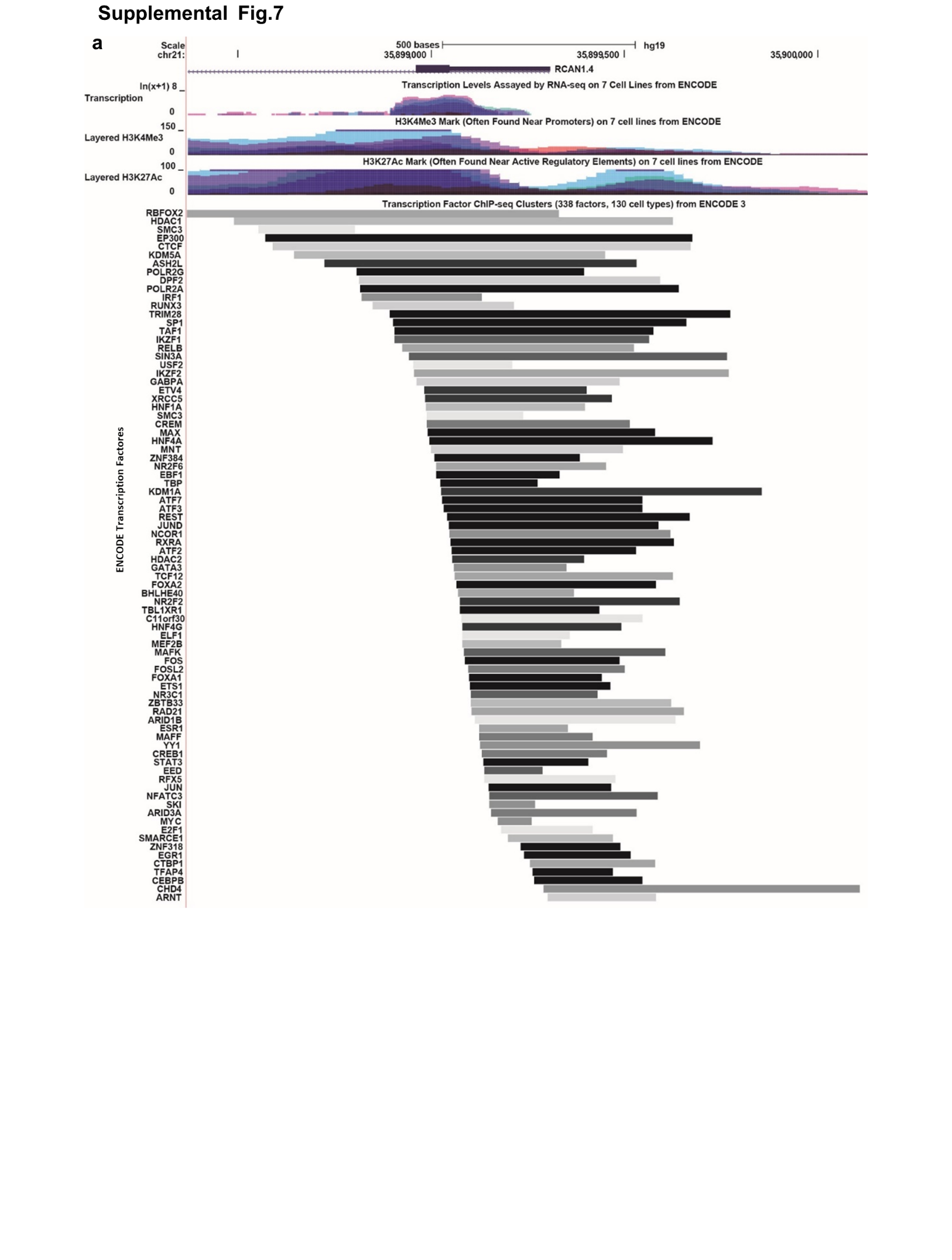
**

**
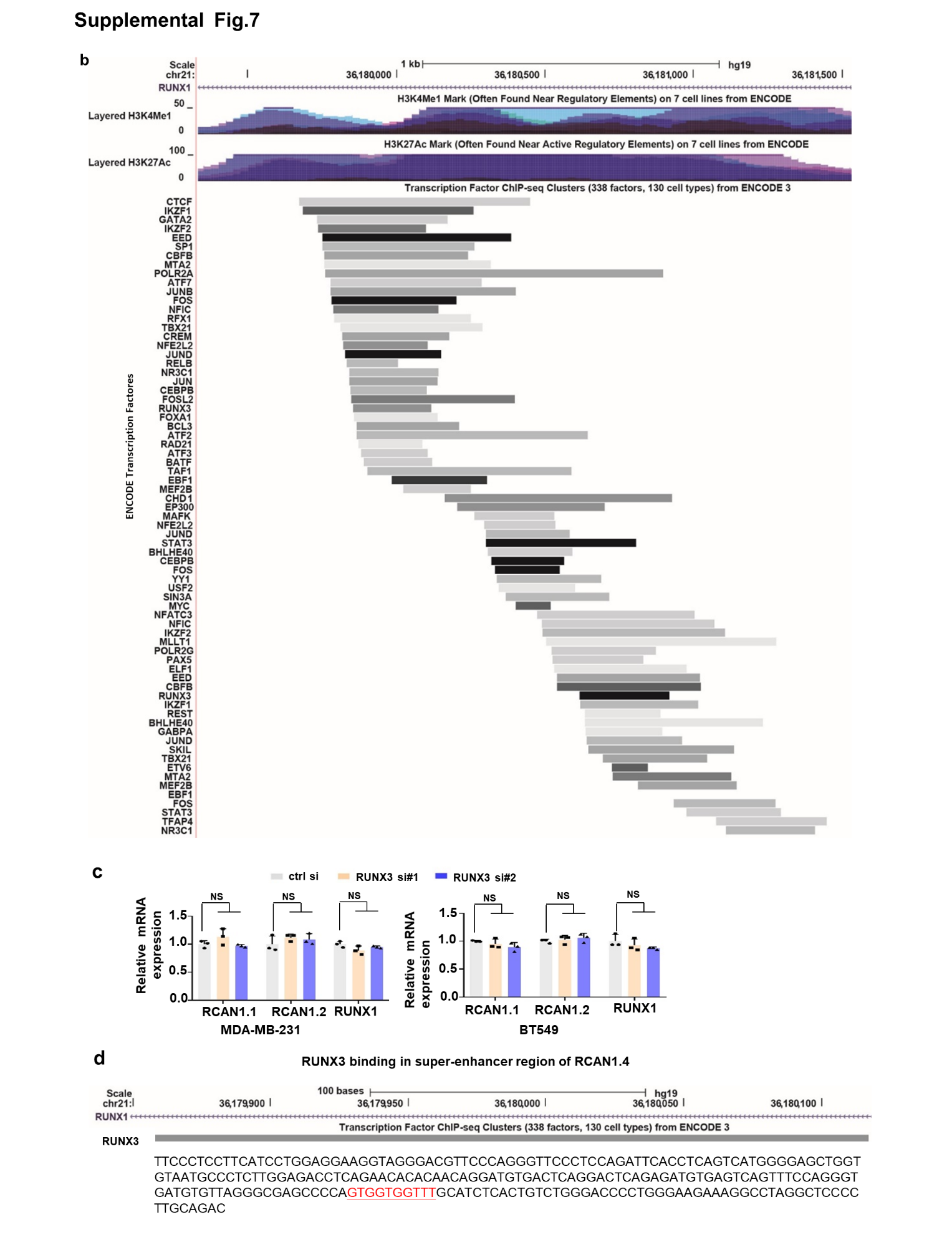
Figure S7.** **RUNX3 activates the transcription of RCAN1.4 by binding to its specific SE. (a-b)** Promoter region (a) / E3 super-enhancer region (b) of RCAN1.4 showing H3K27 acetylation and binding sites for selected ENCODE transcription factors up to seven cell lines (including HSMM, HUVEC, NHEK, NHLF，GM12878, H1-hESC and K562 cell lines) by the UCSC Genome Browser. Transcription factors are color coded in grayscale; the darkness of the box is proportional to maximum ChIP data value seen in any cell line in the region. H3K27ac, H3K4me3 and H3K4me1 data are layered, so some colors may not be reflected in the legend. More information may be found on the UCSC Genome Browser. **(c)** MDA-MB-231 cells and BT549 cells were transfected with siRNAs targeting RUNX3. The RCAN1.1, RCAN1.2 and RUNX1 mRNA levels were quantified using qRT–PCR. Error bars represent mean ± SD, n=3 biological independent samples. NS, not significance. The *P* value was determined by one-way analysis ANOVA with Dunnett’s multiple comparisons test, no adjustments were made for multiple comparisons. Data were representative of three independent experiments. **(d)** RUNX3 binding motif analysis of the super-enhancer region of RCAN1.4 using the JASPAR databases (http://jaspar.genereg.net/). Red highlighted was predicated RUNX3 binding motif.


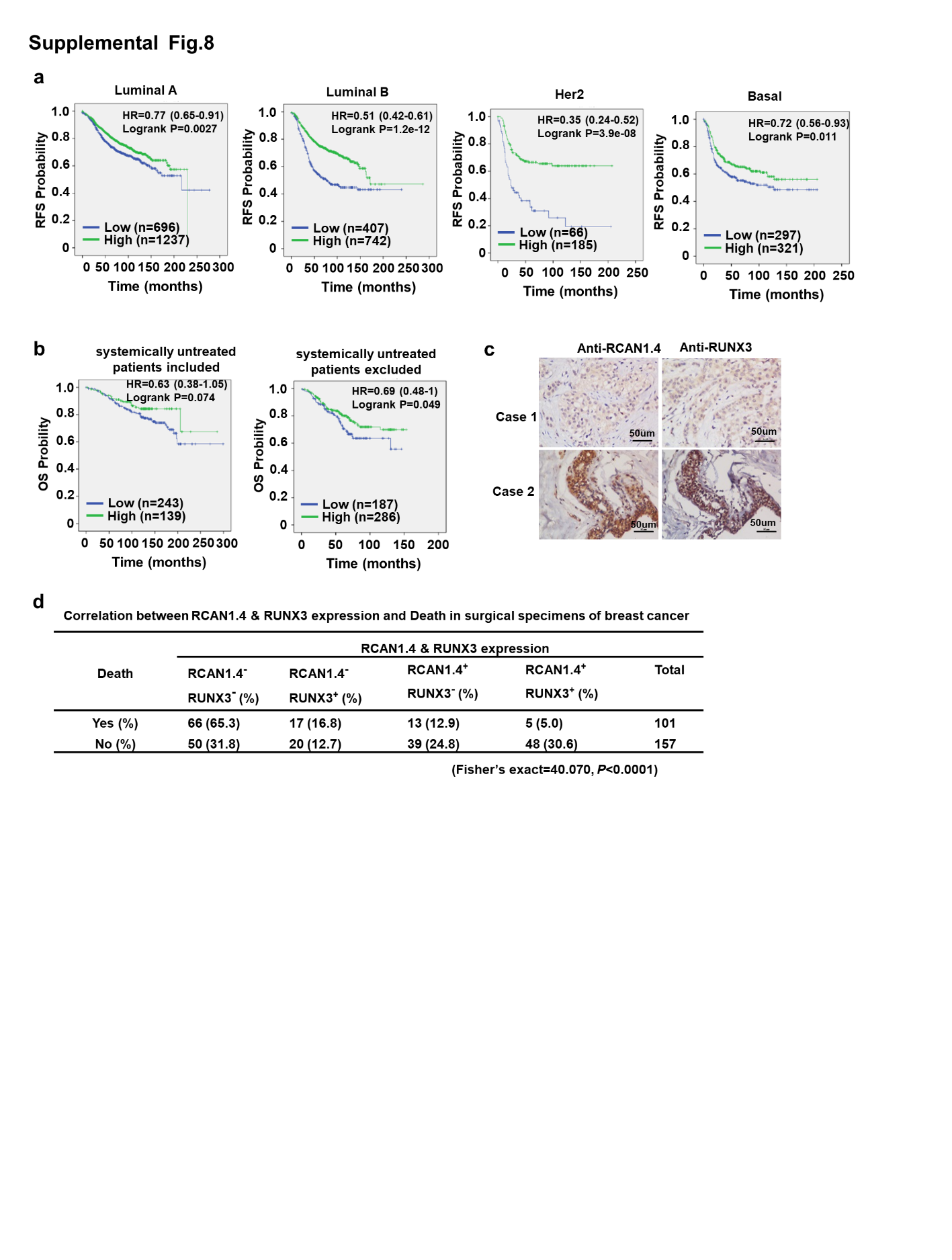


**Figure S8.** **RUNX3 is associated with unfavorable prognosis in BC patients. (a)** Kaplan-Meier analyses of RFS based on RUNX3 (204197_at) mRNA levels were performed by using the KM-plotter breast cancer database (<http://kmplot.com/analysis>). Auto select best cutoff was chosen in the analysis. The patients were stratified according intrinsic subtype as indicated. **(b)** Kaplan-Meier analyses of OS based on RUNX3 (204197_at) mRNA levels were performed by using the KM-plotter breast cancer database (<http://kmplot.com/analysis>). Auto select best cutoff was chosen in the analysis. The patients were stratified according the systemically untreated patients included or excluded.  **(c)**The representative images for RUNX3 staining in two patients with RCAN1.4 expression. Case 1 showed low expression of RCAN1.4 with low expression of RUNX3 staining. Case 2 showed high expression of RCAN1.4 expression with high expression of RUNX3 staining. **(d)** Correlation between RCAN1.4 & RUNX3 expression and Death in breast cancer patients. The *P* value was assessed using the fisher's exact test.

**Figure S9.** **Full unedited Western blotting gels for all figures**

**
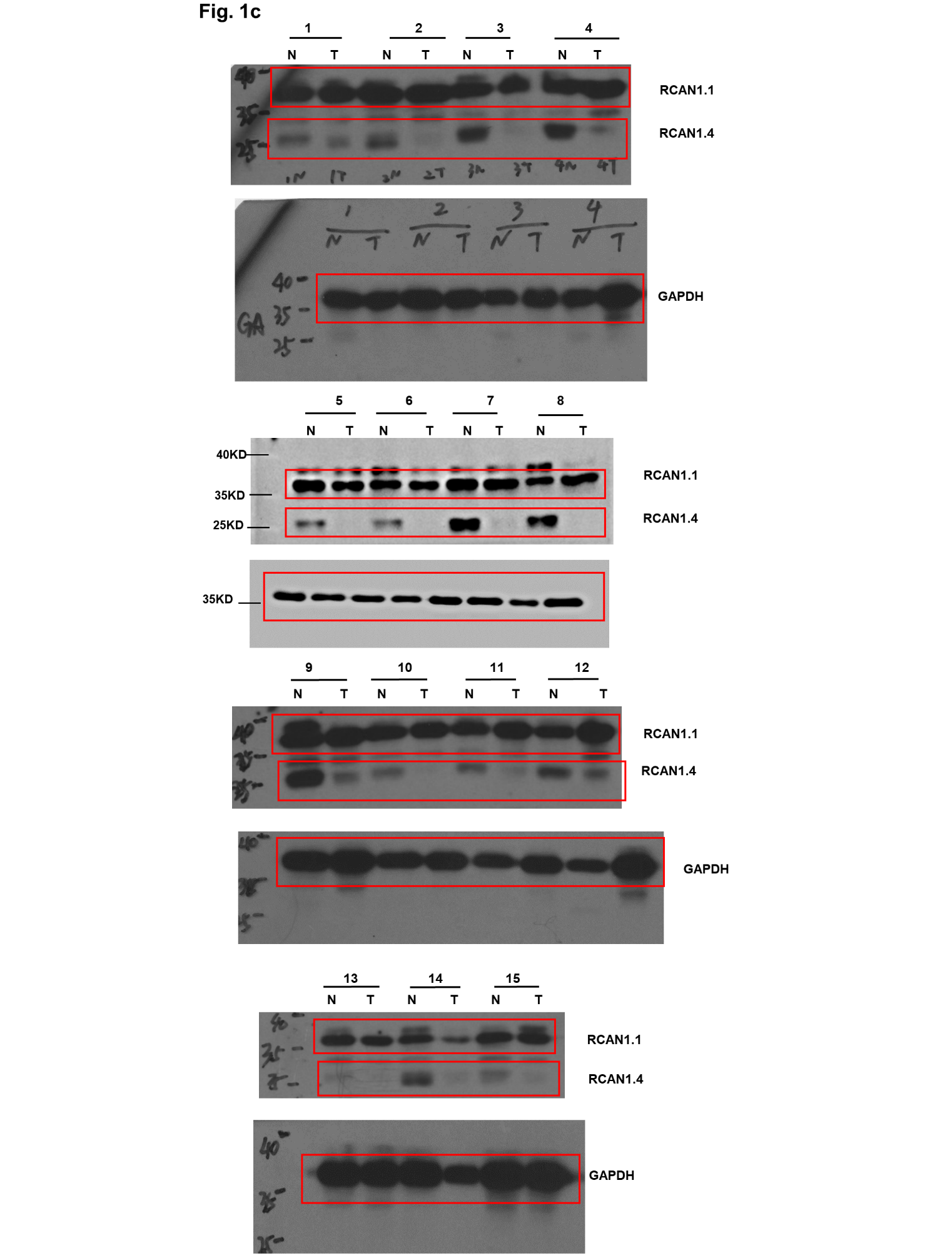
**

**
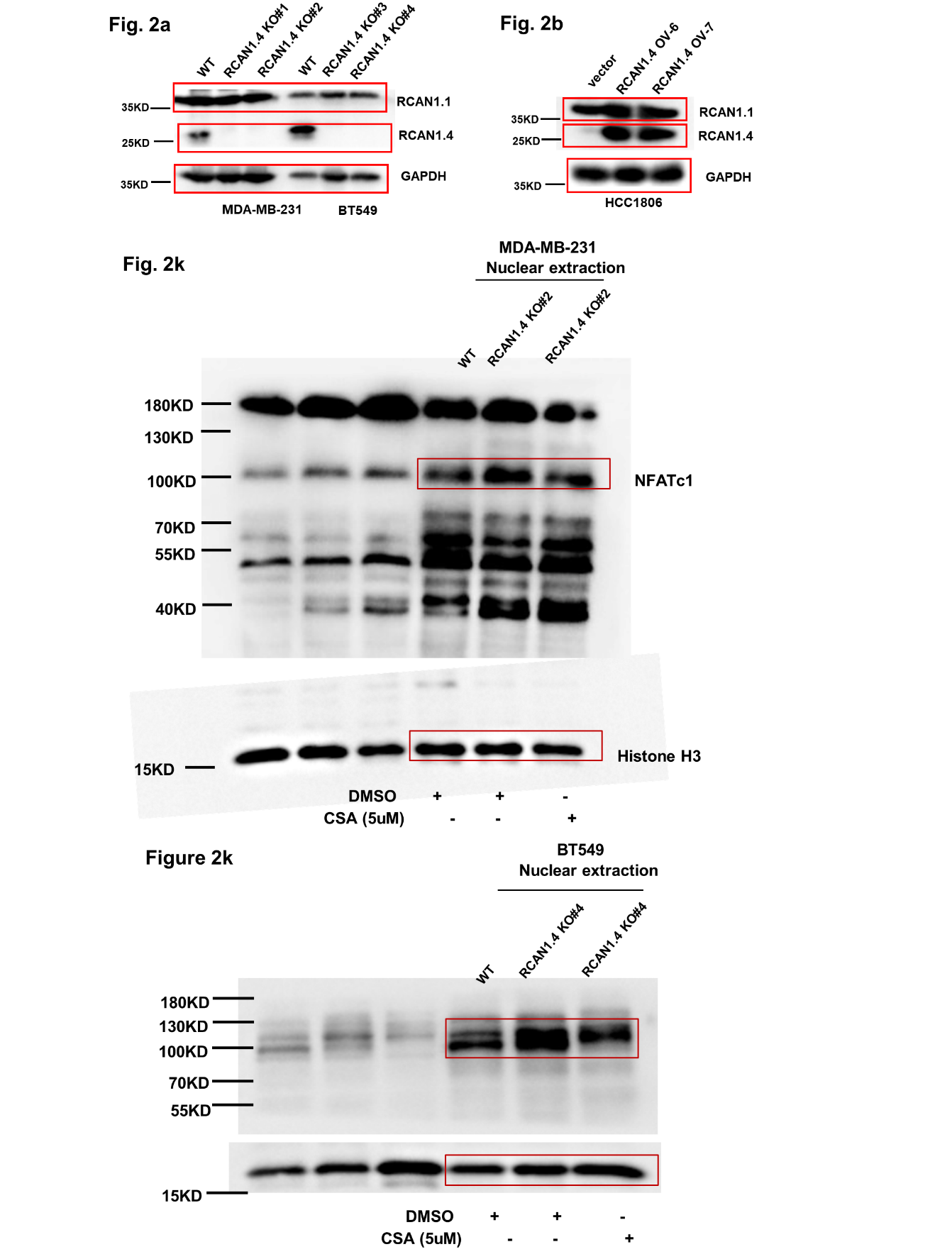
**

**
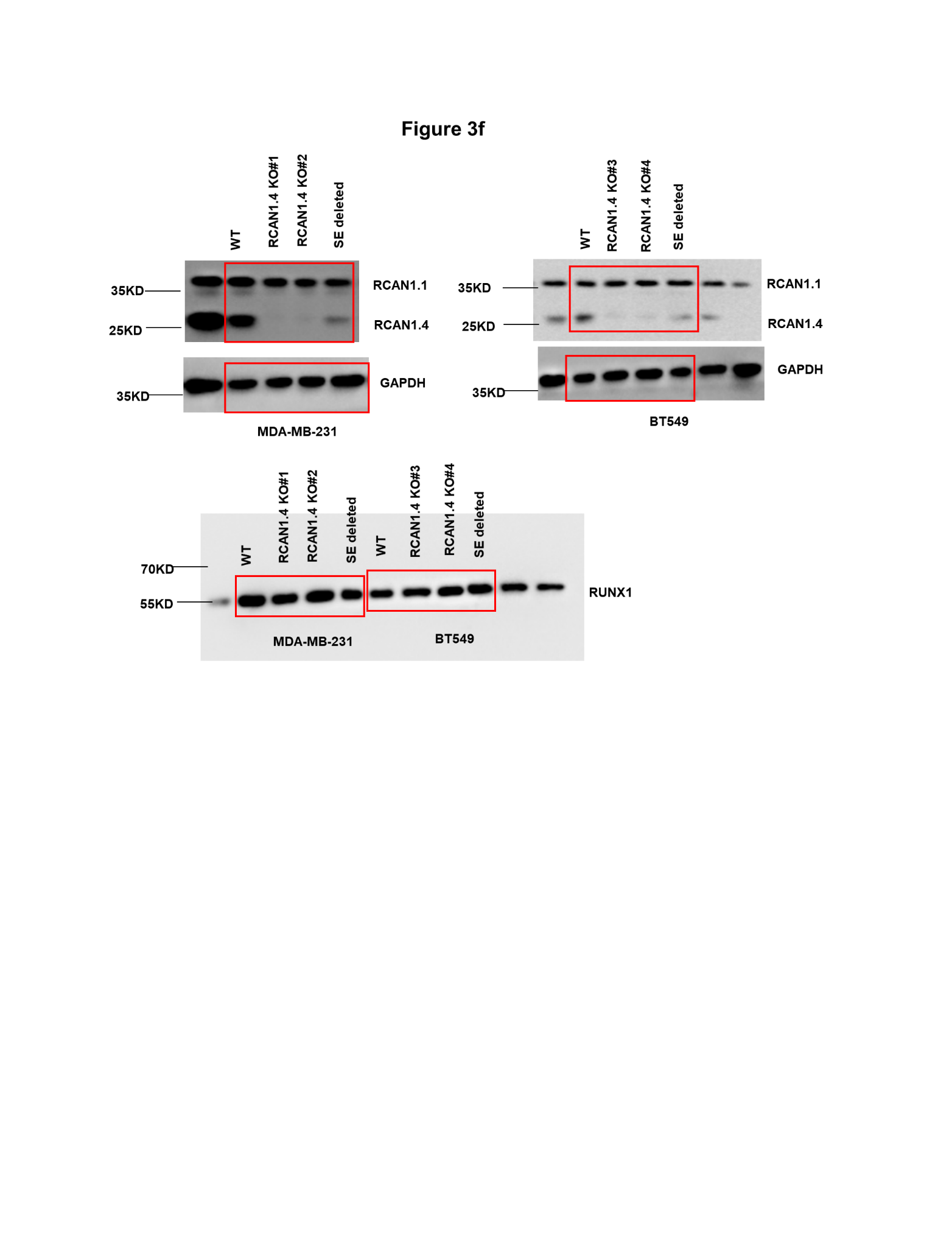
**

**
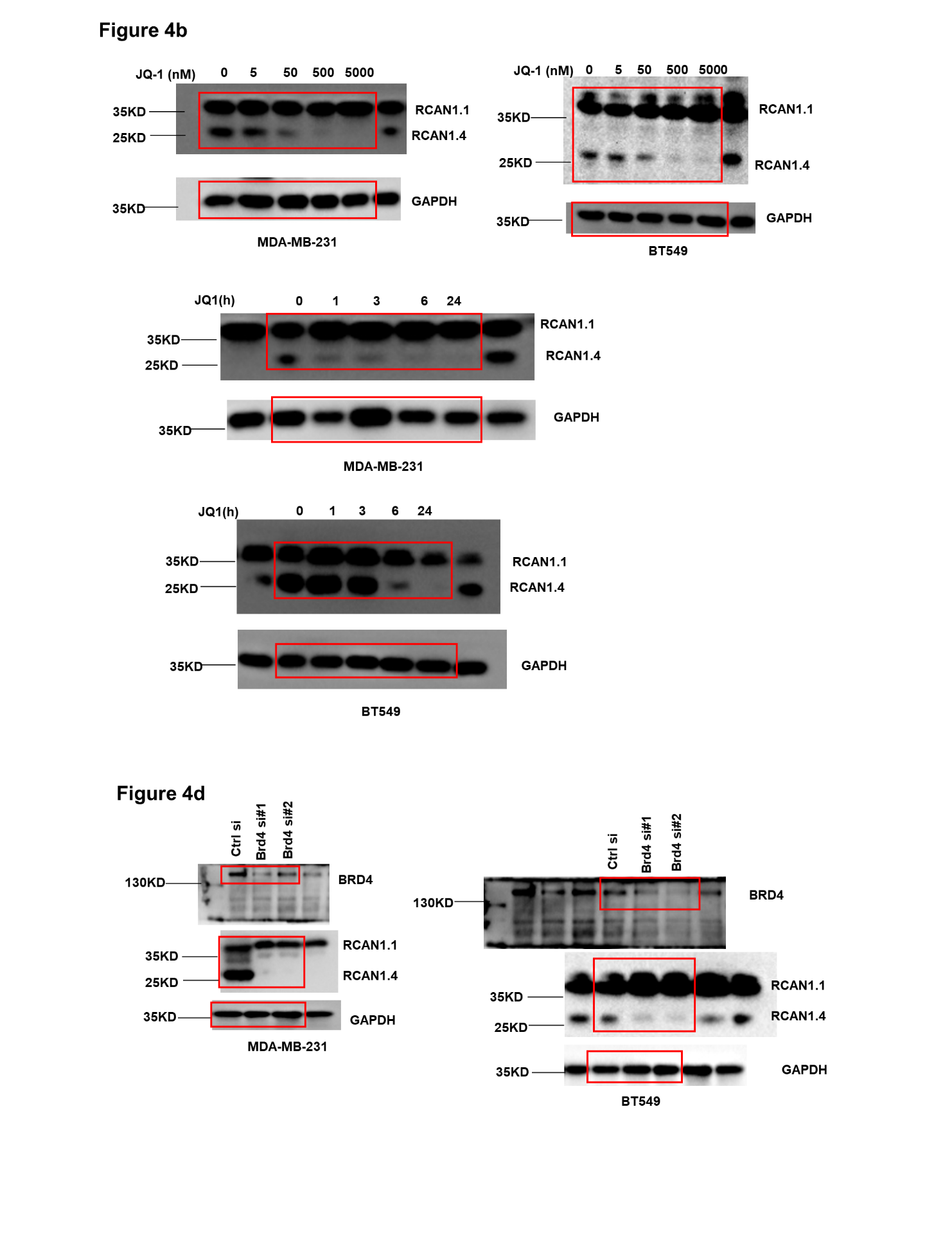
**

**
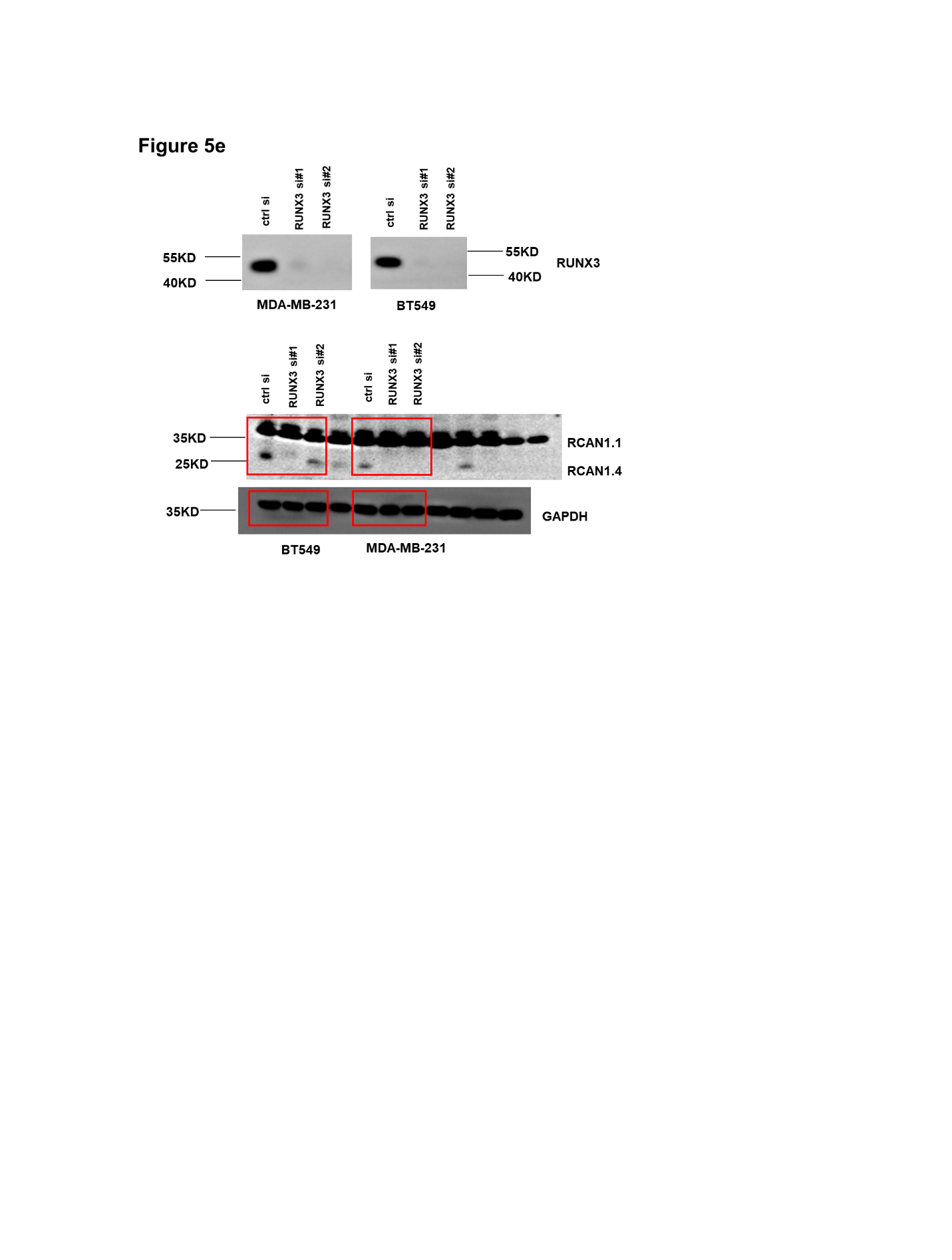
**

**
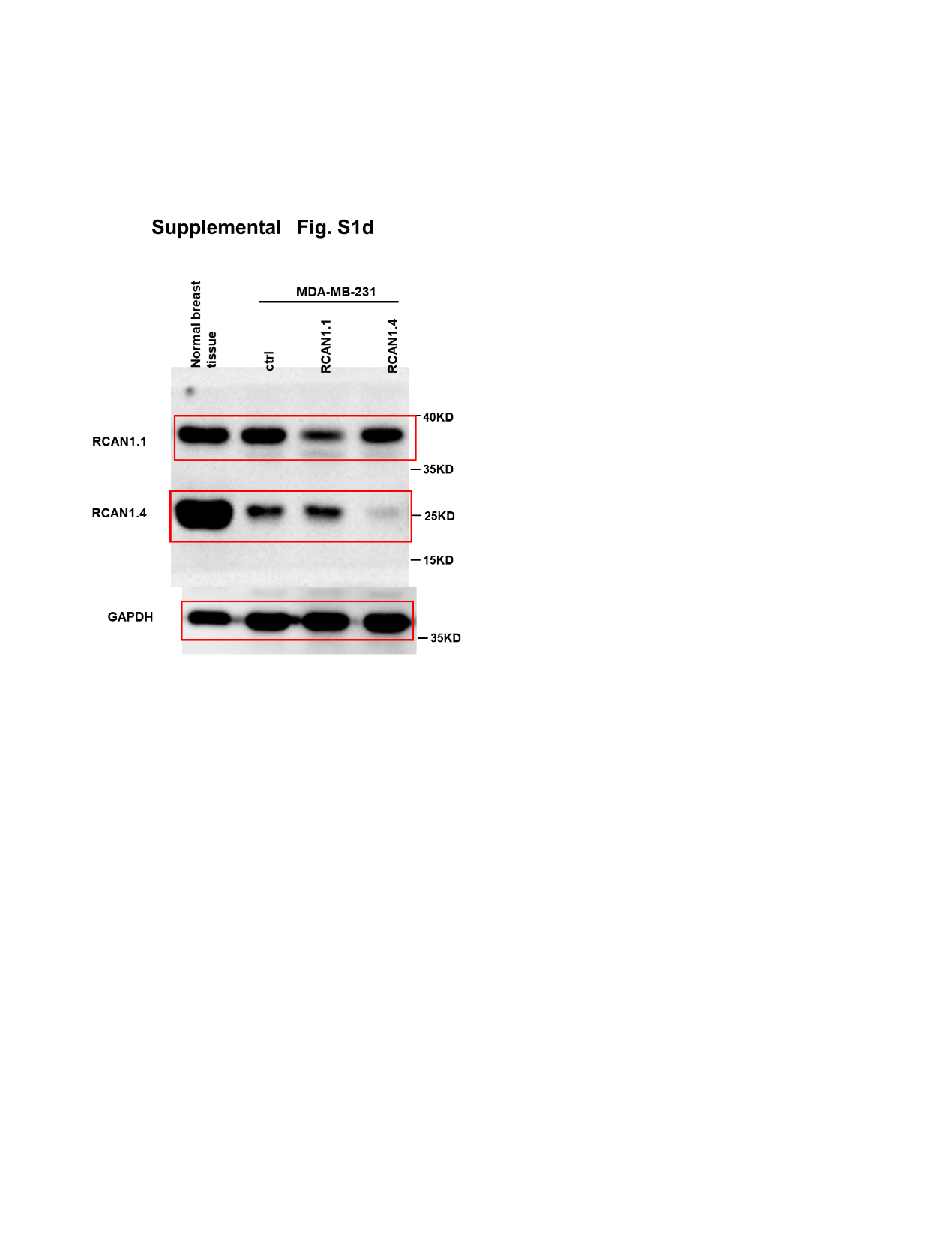
**

**
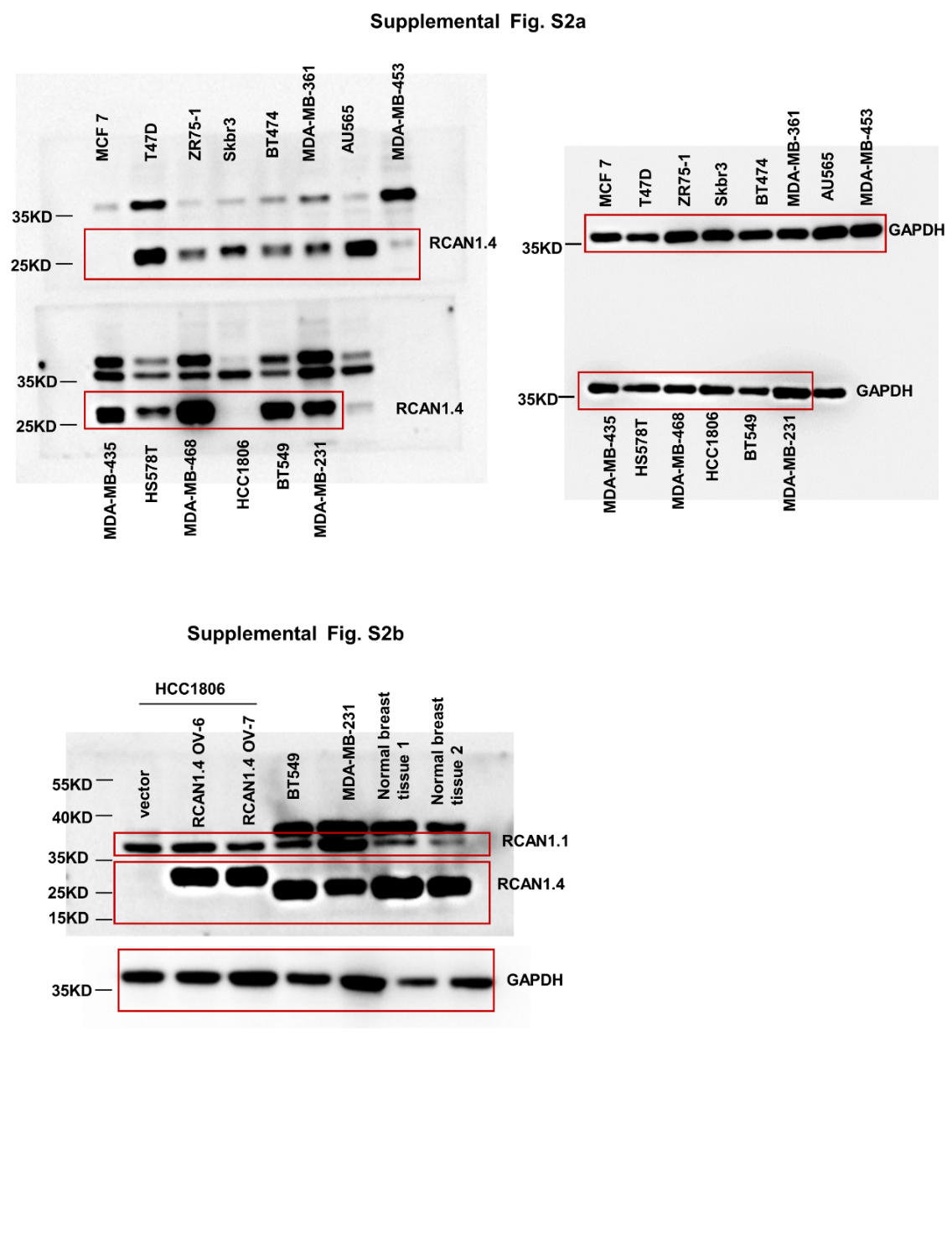
**

**
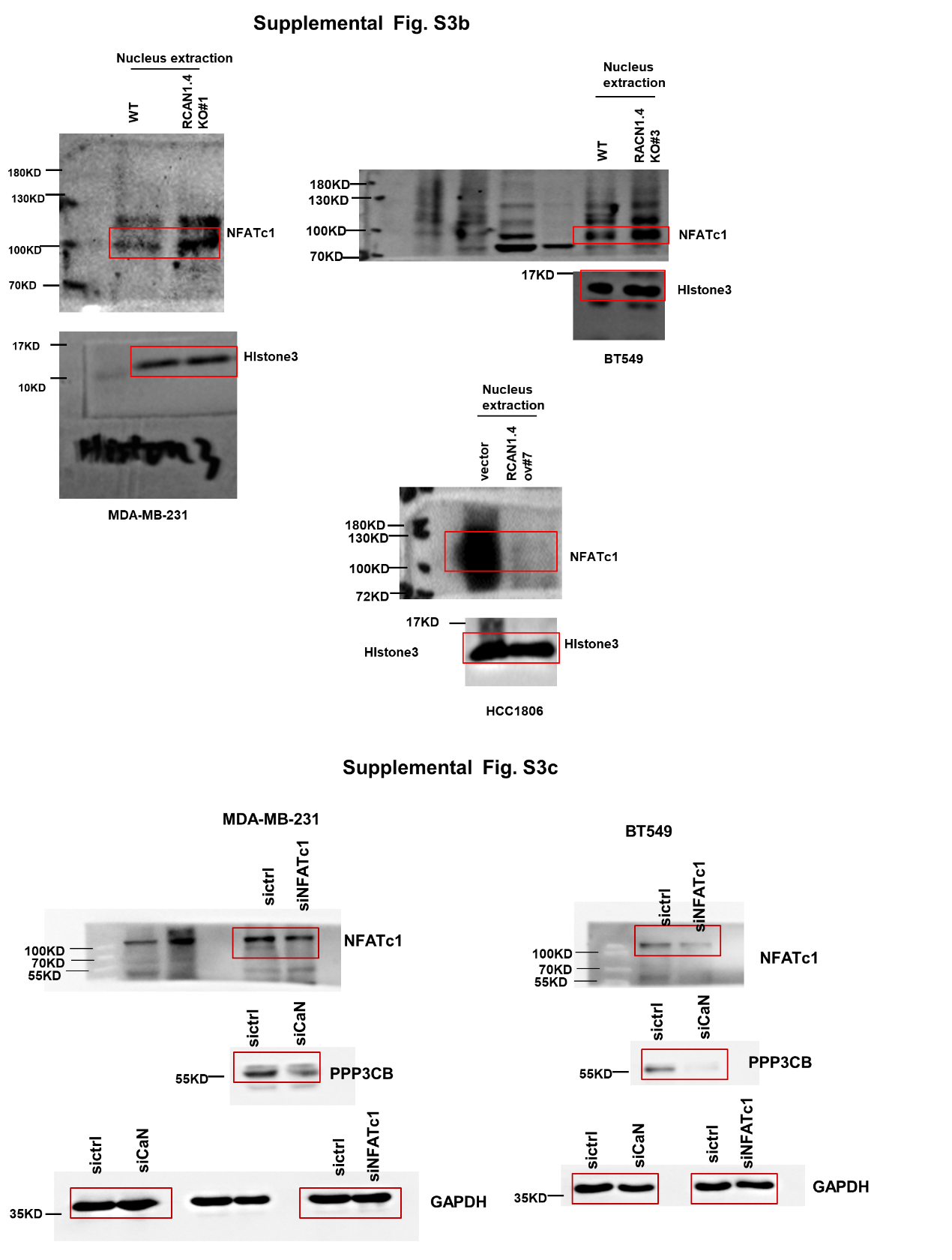
**
